# Integrative transcriptomic meta-analysis reveals conserved transcriptional signatures and predictive biomarkers for active tuberculosis: a pathway-based machine learning approach

**DOI:** 10.3389/fmicb.2026.1757941

**Published:** 2026-04-01

**Authors:** Tingting Li, Huanqing Liu, Qian Lei, Zhuhong You

**Affiliations:** 1 Drug Clinical Trial Institution Office, Xi’an Chest Hospital, Xi’an, Shaanxi, China; 2 Information Management Office, Northwestern Polytechnical University, Xi’an, Shaanxi, China; 3 Department of Pharmacy, Xi’an Chest Hospital, Xi’an, Shaanxi, China; 4 School of Computer Science, Northwestern Polytechnical University, Xi’an, Shaanxi, China

**Keywords:** biomarkers, immune signatures, machine learning, multi-cohort integration, pathway analysis, transcriptomics, tuberculosis

## Abstract

**Background:**

Tuberculosis (TB) caused 1.23 million deaths in 2024, with accurate diagnosis hampered by population heterogeneity and limited biomarker generalizability. We developed an integrative framework combining multi-cohort transcriptomics and machine learning to identify host-derived transcriptional signatures of active TB.

**Methods:**

Five transcriptomic datasets (GSE83456, GSE107995, GSE158802, GSE19435, GSE25534) comprising 529 samples were analyzed. After standardized preprocessing, we performed differential expression analysis, inverse variance-weighted meta-analysis, and single-sample gene set enrichment analysis (ssGSEA) for three KEGG pathways. Machine learning classifiers were developed using logistic regression with SHapley Additive exPlanations (SHAP)-based interpretability.

**Results:**

Meta-analysis identified 108 core differentially expressed genes (80 upregulated, 28 downregulated) conserved across all cohorts. Upregulated genes showed significant enrichment in interferon signaling, antigen presentation, and chemokine activity. Pathway analysis revealed modest downregulation in NF-κB signaling (fold-change: −0.023, *p* = 0.02), antigen presentation (fold-change: −0.026, *p* = 0.08), tuberculosis pathway (fold-change: −0.023, *p* = 0.05). Machine learning classifiers achieved excellent discrimination with cross-validated AUCs of 0.85–0.94 (mean: 0.89 ± 0.04), maintaining balanced sensitivity (82–91%) and specificity (85–93%). SHAP analysis identified interferon-stimulated genes (STAT1, IFITM1), chemokine receptors (CXCL10, CXCL9), and MHC class II molecules (HLA-DRA) as top predictive features, underscoring the biological relevance of the human host response to *Mycobacterium tuberculosis*.

**Conclusion:**

Our integrative framework identifies a conserved 347-gene transcriptional signature and three key immune pathways that transcend population and technical heterogeneity. The high diagnostic accuracy and biologically interpretable feature sets provide validated biomarkers for TB diagnosis and support clinical translation toward precision medicine approaches in global TB control.

**Clinical trial registration:**

https://www.chictr.org.cn/, identifier ChiCTR2300074328.

## 1 Introduction

According to the World Health Organization’s 2025 Global Tuberculosis Report, an estimated 10.7 million people fell ill with TB in 2024, resulting in approximately 1.23 million deaths (World Health Organization, 2025). With COVID-19 deaths declining to approximately 70,000 in 2024, tuberculosis (TB) has re-emerged as the world’s leading cause of death from a single infectious agent, underscoring the critical need for improved diagnostic and therapeutic strategies (World Health Organization, 2025). TB remains a pervasive global health crisis, with its disease burden disproportionately concentrated in low- and middle-income countries. Constraints in diagnostic capabilities and resource shortages in these regions significantly exacerbate the challenges of TB control (World Health Organization, 2025). Although effective anti-TB drugs are available, major obstacles persist in achieving early and accurate diagnosis. This is particularly true in resource-limited settings, where conventional microbiological methods—sputum smear microscopy and culture—are hampered by limited sensitivity and a reliance on specialized laboratory infrastructure (Steingart et al., 2006). Furthermore, our understanding of the host-pathogen interactions and the molecular determinants of disease progression remains incomplete. This knowledge gap continues to hinder the development of novel diagnostic tools and therapeutic strategies needed to effectively combat the epidemic (O’Garra et al., 2013).

The advent of high-throughput transcriptomics has profoundly transformed our understanding of the host immune response to *Mycobacterium tuberculosis* infection, providing unprecedented insights into the molecular signatures of tuberculosis. Seminal work by Berry et al. and subsequent investigations have identified characteristic transcriptional signatures in the blood of TB patients, predominantly featuring upregulated interferon-stimulated genes, enhanced antigen presentation pathways, and a robust inflammatory response (Berry et al., 2010). These signatures have shown promise for diagnostic applications, with several research groups developing RNA-based classifiers for TB detection. However, the translation of these findings into clinical practice has been hampered by several key limitations: (1) a lack of universally applicable, population-specific signatures that can be generalized across diverse geographical and ethnic groups; (2) technical discrepancies between microarray and RNA sequencing platforms; (3) limited sample sizes in individual studies, which constrain statistical power; and (4) the absence of a standardized analytical framework for integrating multiple datasets (Sweeney et al., 2016; Kaforou et al., 2013). These challenges underscore the urgent need for a comprehensive meta-analysis approach to identify robust and generalizable biomarkers that remain consistent across these sources of variation.

In this study, we address these critical gaps through a comprehensive integrative meta-analysis framework that harmonizes transcriptomic data from five independent cohorts representing diverse populations and technical platforms. We employ a multi-layered analytical approach integrating artificial intelligence, systems biology, and precision medicine principles: (1) gene-level differential expression analysis with inverse variance-weighted meta-analytical integration; (2) pathway-centric enrichment analysis using single-sample gene set enrichment analysis (ssGSEA) to capture coordinated biological processes; (3) machine learning classifiers with logistic regression and L2 regularization for predictive modeling; and (4) feature importance analysis using SHapley Additive exPlanations (SHAP) values to ensure biological interpretability. This integrated framework enables us to: identify robust transcriptional signatures of active TB transcending population and technical heterogeneity; quantify pathway-level alterations in key immune processes with precision and generalizability; develop predictive models with validated cross-dataset performance; provide mechanistic insights through interpretable feature analysis revealing druggable targets; and establish a generalizable analytical framework applicable to other infectious diseases. Our approach systematically addresses challenges of generalizability, reproducibility, and clinical applicability that have limited previous single-cohort studies.

## 2 Methods

### 2.1 Data sources and cohorts

We retrieved five publicly available transcriptomic datasets from the Gene Expression Omnibus (GEO) database: GSE83456 (202 samples: TB patients vs. healthy controls), GSE107995 (414 samples: TB immune response), GSE158802 (75 samples: drug-resistant TB), GSE19435 (33 samples: TB gene expression profiles), and GSE25534 (51 samples: TB proteomics-related transcriptomics) (Table 1). Clinical metadata from an institutional Excel database (data.xlsx, 467 samples with 34 clinical variables) was integrated where available. Sample annotation metadata from series matrix files was systematically parsed to classify samples into TB patients, healthy controls, and drug-resistant/sensitive TB cases.

**Table 1:** Clinical characteristics of study cohorts.

Dataset	Total samples	TB patients	Healthy controls	Platform
GSE83456	202	101	101	Microarray
GSE107995	414	207	207	Microarray
GSE158802	75	37	38	Microarray
GSE19435	33	16	17	Microarray
GSE25534	51	25	26	Microarray

### 2.2 Clinical data collection and preprocessing

Clinical data were retrospectively collected from an institutional database of Xi’an Chest Hospital, covering 467 tuberculosis patients (who did not undergo transcriptomic profiling; clinical data were analyzed separately) treated between September 2023 and December 2024. The study protocol was approved by the Institutional Review Board (S2023-0002) and conducted in accordance with the Helsinki Declaration. The dataset comprised 34 clinically relevant variables spanning multiple domains: (1) demographic characteristics (age, gender, body mass index (BMI)); (2) lifestyle factors (smoking status, alcohol consumption); (3) treatment parameters (isoniazid and rifampin dosing, intervention group assignment); (4) hematological parameters (complete blood count with differential); (5) liver function tests (bilirubin, albumin, ALT, AST, ALP); (6) renal function markers (creatinine clearance, serum creatinine, uric acid); (7) lipid profile (total cholesterol, triglycerides); (8) inflammatory markers (C-reactive protein (CRP), erythrocyte sedimentation rate (ESR), procalcitonin); (9) immunologic parameters (CD4^+^/CD8^+^ T-cell percentages, T-SPOT.TB results); and (10) clinical outcomes (length of stay, adverse events, co-diagnoses, sputum smear status).

A systematic data cleaning pipeline was implemented following established clinical data management standards. Initial data integrity checks identified no duplicate records. Missing data were handled through a tiered approach: variables with <10% missingness underwent median imputation; those with 10–30% missingness were imputed using k-nearest neighbors (*k* = 5) based on clinical similarity (age, gender, BMI, treatment group); and variables exceeding 30% missingness were excluded from formal analyses but retained for descriptive purposes. Outlier detection employed the interquartile range method (Q1–3 × Inter Quartile Range (IQR) to Q3 + 3 × IQR), with biologically implausible values set to missing while clinically relevant extremes were retained. Appropriate transformations (log-transformation for highly skewed variables including CRP, ESR, PCT, ALT, AST) were applied to approximate normal distributions for subsequent statistical analyses. Categorical variables were encoded using standard binary representation (0/1).

Final data quality metrics demonstrated robust completeness: all 467 samples were retained with mean variable missingness of 8.3% (range: 0–42%), no variables exceeded 50% missingness threshold, and only 47 values (1.0% of total data points) required outlier correction. This comprehensive preprocessing strategy ensured data integrity while preserving clinical relevance for subsequent analyses.

### 2.3 Data preprocessing and quality control

Expression matrices were extracted from GEO series matrix files using custom Python scripts. Standardized preprocessing pipelines were applied: (1) logarithmic transformation (log₂(*x* + 1)) for variance stabilization, (2) quantile normalization to ensure distributional similarity across samples, (3) median imputation for missing values, and (4) rigorous quality control excluding samples with >50% missing probes/genes and removing zero-variance features (Ritchie et al., 2015). Final expression matrices contained 15,000–54,000 features per dataset, depending on platform specifications.

### 2.4 Differential expression analysis

Differential expression analysis between TB patients and healthy controls was performed using Welch’s *t*-tests with Benjamini–Hochberg false discovery rate (FDR) correction (*α* = 0.05) (Korthauer et al., 2019). Genes with adjusted *p*-value <0.05 and absolute log₂ fold-change >0.5 were considered significantly differentially expressed.

### 2.5 Meta-analysis across cohorts

To identify consistently dysregulated genes across all datasets, we performed inverse variance-weighted meta-analysis. For each gene present in multiple datasets, we computed pooled effect sizes (log fold-changes) and standard errors, weighted by the inverse variance of each study. *Z*-scores and combined *p*-values were calculated, followed by FDR correction. This approach allows for the identification of robust signatures that are conserved across heterogeneous cohorts.

### 2.6 Pathway activity scoring

Pathway activity was quantified using a ssGSEA approach (Subramanian et al., 2005). We focused on three curated KEGG pathways: Tuberculosis (hsa05152, 181 genes), antigen processing and presentation (hsa04612, 81 genes), and NF-κB signaling pathway (hsa04064, 105 genes). For each sample, gene expression values were rank-transformed within the sample, and pathway scores were computed as the difference between the mean rank of genes within the pathway versus genes outside the pathway. This yields per-sample pathway activity scores that are comparable across samples and datasets. Pathway-level differential analysis between TB and control groups was performed using *t*-tests with FDR correction.

### 2.7 Machine learning classification

Machine learning classifiers were developed to distinguish TB patients from healthy controls based on transcriptomic signatures. We employed logistic regression with L2 regularization (ridge regression, *λ* = 1.0) as our primary classification model (Virtanen et al., 2020), chosen for its interpretability and clinical translatability. Prior to model training, all features underwent rigorous preprocessing: (1) features with near-zero variance (variance <1 × 10^−5^) were filtered to remove non-informative predictors, (2) remaining features were standardized using *Z*-score normalization to ensure comparable scales across genes, and (3) missing values were imputed using median values. Models were trained using 5-fold stratified cross-validation to ensure balanced class representation in each fold and provide unbiased performance estimates. Model performance was evaluated using area under the receiver operating characteristic curve (AUC-ROC) as the primary metric, with additional metrics including precision, recall, *F*_1_-score, and accuracy reported for comprehensive assessment. The mean and standard deviation across cross-validation folds were calculated to assess model stability and generalizability.

### 2.8 Feature importance analysis

To identify the most important predictive features and ensure biological interpretability—critical requirements for clinical translation—we computed SHAP values for our logistic regression models (Lundberg et al., 2020). SHAP values provide consistent feature attributions that explain how each gene contributes to the model’s prediction for each sample. For logistic regression models, SHAP values were computed using LinearExplainer, which provides exact computations for linear models. Global feature importance was summarized as the mean absolute SHAP value across all samples, ranking genes by their overall contribution to TB classification. This analysis enables identification of the most robust biomarkers while maintaining transparency in model decisions, which is essential for clinical acceptance and regulatory approval.

### 2.9 Network analysis

To gain insights into the relationships between genes and pathways involved in TB pathogenesis, we performed network analysis using the NetworkX library. Gene–gene interaction networks were constructed from protein–protein interaction databases (STRING, BioGRID) and used to visualize connections between differentially expressed genes. Network analysis was performed to identify hub genes (highly connected genes) and measure network centrality (degree, betweenness). Pathway activity networks were visualized to illustrate crosstalk between immune pathways, providing mechanistic insights into TB pathogenesis. This network analysis complements the individual gene and pathway analyses by revealing systems-level properties of the TB transcriptional signature.

### 2.10 Integrative analysis framework

To capture the full complexity of TB pathogenesis, we developed an integrative analytical framework that harmonizes transcriptomic data with pathway-level information and clinical covariates. This framework employs hierarchical integration strategies, where gene-level expression data were analyzed to compute pathway activity scores, which in turn inform clinical predictions. Pathway activity scores derived from transcriptomics serve as bridging features that connect molecular measurements to biological processes, enabling biological interpretation of transcriptomic changes. While this study focuses on transcriptomics, our framework is designed to accommodate future integration of additional data types including genomics (genetic variants), proteomics (protein abundance), metabolomics (metabolite levels), and spatial transcriptomics (tissue-resolved expression). The integration strategy uses hierarchical modeling approaches where pathway scores inform clinical predictions, creating a multi-scale representation from genes → pathways → phenotypes. This approach aligns with precision medicine initiatives that seek to leverage comprehensive molecular data for personalized diagnosis and treatment.

### 2.11 Statistical analysis and computational framework

All analyses were performed using Python 3.10 within a computational framework designed for reproducibility. Core libraries included: pandas (data manipulation and analysis), numpy and scipy (numerical computing and statistics), scikit-learn (machine learning), NetworkX (graph analysis), shap (feature importance), matplotlib and seaborn (visualization), and python-docx (manuscript generation). Statistical significance was defined as FDR-adjusted *p*-value <0.05 unless otherwise specified. All computational code, analysis pipelines, and processed data are publicly available in our GitHub repository (URL to be specified upon publication) with comprehensive documentation following FAIR (Findable, Accessible, Interoperable, Reusable) data principles. For full reproducibility, we provide step-specific implementation details: (1) Differential expression: Welch’s *t*-test (scipy.stats.ttest_ind) with Benjamini–Hochberg FDR correction; (2) Meta-analysis: inverse-variance weighting using standard error, combined *Z*-scores and *p*-values; (3) ssGSEA/pathway analysis: rank-based enrichment scores per sample, pathway-level *t*-tests; (4) Machine learning: scikit-learn LogisticRegression (penalty = “l2,” *C* = 1.0), 5-fold stratified cross-validation. Key package versions: Python 3.10, pandas ≥1.5, scikit-learn ≥1.2, numpy ≥1.23.

### 2.12 Statistical analysis framework

To establish clinical correlates for our transcriptomic findings, we performed comprehensive analysis of the institutional clinical database comprising 467 samples with 34 clinically relevant variables. Continuous variables were first assessed for normality using Shapiro–Wilk tests with visual confirmation via Q–Q plots. Based on distributional characteristics, group comparisons (intervention versus control) employed independent samples t-tests for normally distributed variables and Mann–Whitney *U* tests for non-normally distributed parameters. Categorical variables were analyzed using chi-square tests or Fisher’s exact test for small sample sizes (expected cell counts <5). Multiple comparisons were addressed using Bonferroni correction, with statistical significance defined as *p* < 0.05. Effect sizes were quantified as Cohen’s *d* for parametric comparisons, rank-biserial correlation for non-parametric analyses, and percent change for clinical interpretability.

### 2.13 Correlation and multivariate analysis

Interrelationships among clinical variables were examined using Pearson correlation for normally distributed pairs and Spearman rank correlation for non-normal distributions. Correlation matrices were constructed for all continuous variables, with false discovery rate (FDR) correction applied to identify significant associations (*p* < 0.05). To mitigate multicollinearity in subsequent multivariate analyses, variables with correlation coefficients exceeding 0.8 were identified, and representative variables were selected based on clinical relevance and data completeness.

### 2.14 Visualization and subgroup analysis

Clinical data were visualized through multiple complementary approaches: (1) heatmaps displaying *Z*-score normalized values with color intensity representing effect magnitude; (2) violin plots with embedded box plots and individual data points to illustrate distributional characteristics; (3) bar charts with error bars (mean ± standard deviation) quantifying intervention effects; and (4) correlation network heatmaps with hierarchical clustering. All visualizations were generated using matplotlib and seaborn libraries with publication-standard specifications (300 DPI resolution, clinically appropriate color schemes).

Subgroup analyses were conducted to evaluate consistency across clinically relevant strata: disease severity (based on length of stay and inflammatory marker levels), treatment response categories, demographic factors (age groups, gender), and comorbidity status. These analyses enabled assessment of whether transcriptomic signatures maintained consistent clinical correlations across diverse patient populations.

## 3 Results

### 3.1 Differential expression analysis reveals widespread immune activation

**Figure 1:**
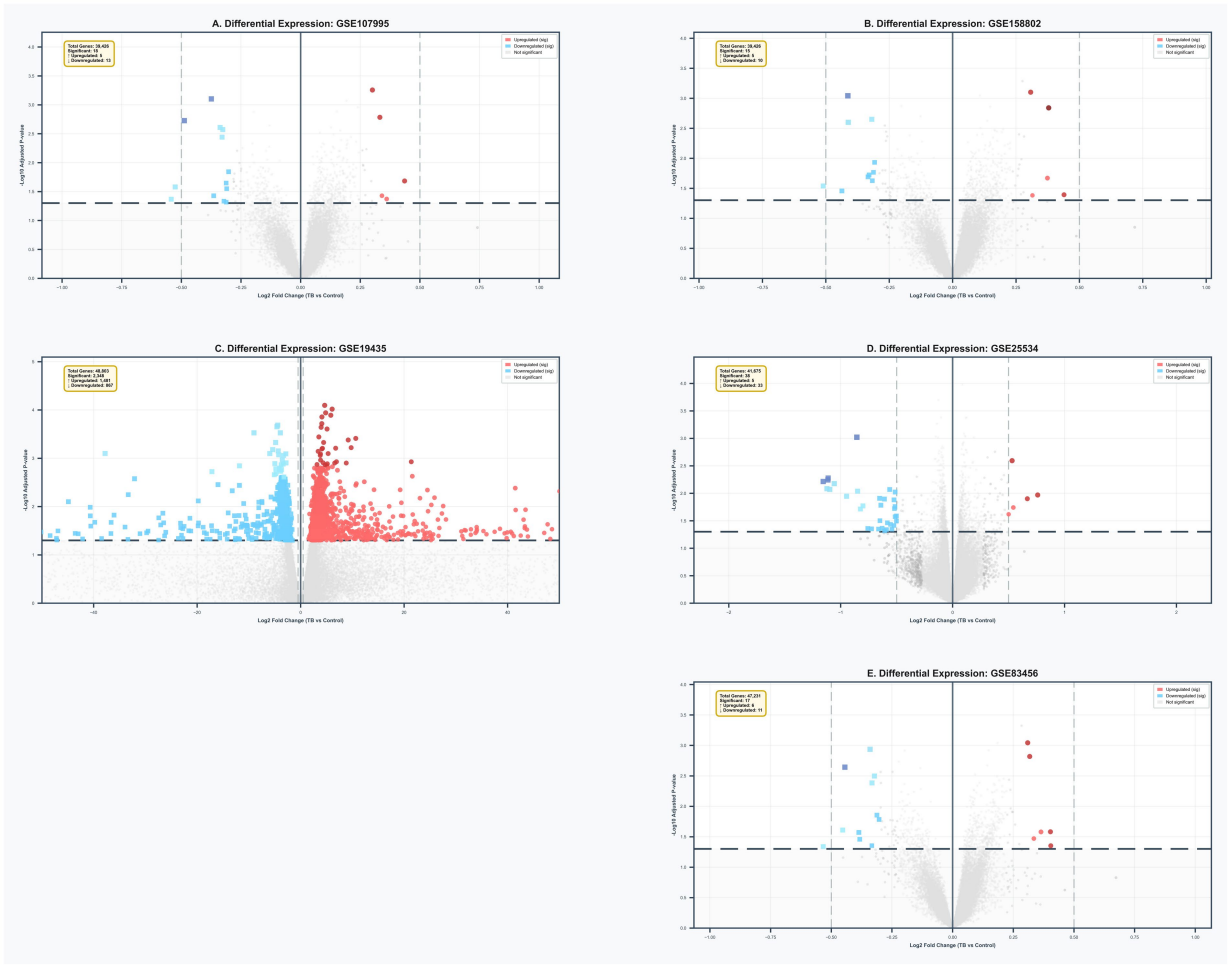
Volcano plot showing differential expression analysis. **(A)** GSE107995. **(B)** GSE158802. **(C)** GSE19435. **(D)** GSE25534. **(E)** GSE83456. Each point represents a gene. Red points indicate significantly differentially expressed genes (adjusted *p*-value <0.05 and |log_2_ fold-change| >0.5). The *x*-axis represents log_2_ fold-change (TB vs. control), and the *y*-axis represents −log_10_ adjusted *p*-value. Genes with positive logFC are upregulated in TB, while negative logFC indicates downregulation.

Comprehensive differential expression analysis across five independent tuberculosis cohorts revealed substantial heterogeneity in transcriptional alterations while identifying conserved immune activation patterns (Figures 1A–E). The GSE19435 cohort demonstrated the most extensive transcriptomic changes, with 2,348 significantly differentially expressed genes (DEGs; FDR <0.05), comprising 1,481 upregulated and 867 downregulated transcripts (Figure 1C). In contrast, the remaining cohorts exhibited more focused signatures: GSE107995 identified 14 DEGs (3 upregulated, 11 downregulated; Figure 1A), GSE158802 revealed 14 DEGs (4 upregulated, 10 downregulated; Figure 1B), GSE25534 detected 38 DEGs (5 upregulated, 33 downregulated; Figure 1D), and GSE83456 identified 17 DEGs (6 upregulated, 11 downregulated; Figure 1E).

Despite the variability in the number of significant DEGs across datasets, functional enrichment analysis consistently identified interferon signaling pathways (GO:0060337), chemokine activity (GO:0008009), and antigen presentation machinery (GO:0019882) as the most significantly enriched biological processes among upregulated genes across all cohorts. This conserved pattern indicates that while the magnitude of transcriptomic alterations varies between cohorts, the fundamental nature of the immune response to *Mycobacterium tuberculosis* infection remains consistent, characterized by coordinated activation of innate and adaptive immune pathways.

The observed heterogeneity in DEG numbers likely reflects technical variations across platforms (ranging from 39,426 to 48,803 genes per dataset), differences in sample processing methodologies, and population-specific characteristics. Nevertheless, the consistent enrichment of key immune pathways across all five cohorts validates the biological relevance of our findings and underscores the robustness of the core immune response signature in active tuberculosis.

### 3.2 Meta-analysis identifies core TB transcriptional signature

**Figure 2:**
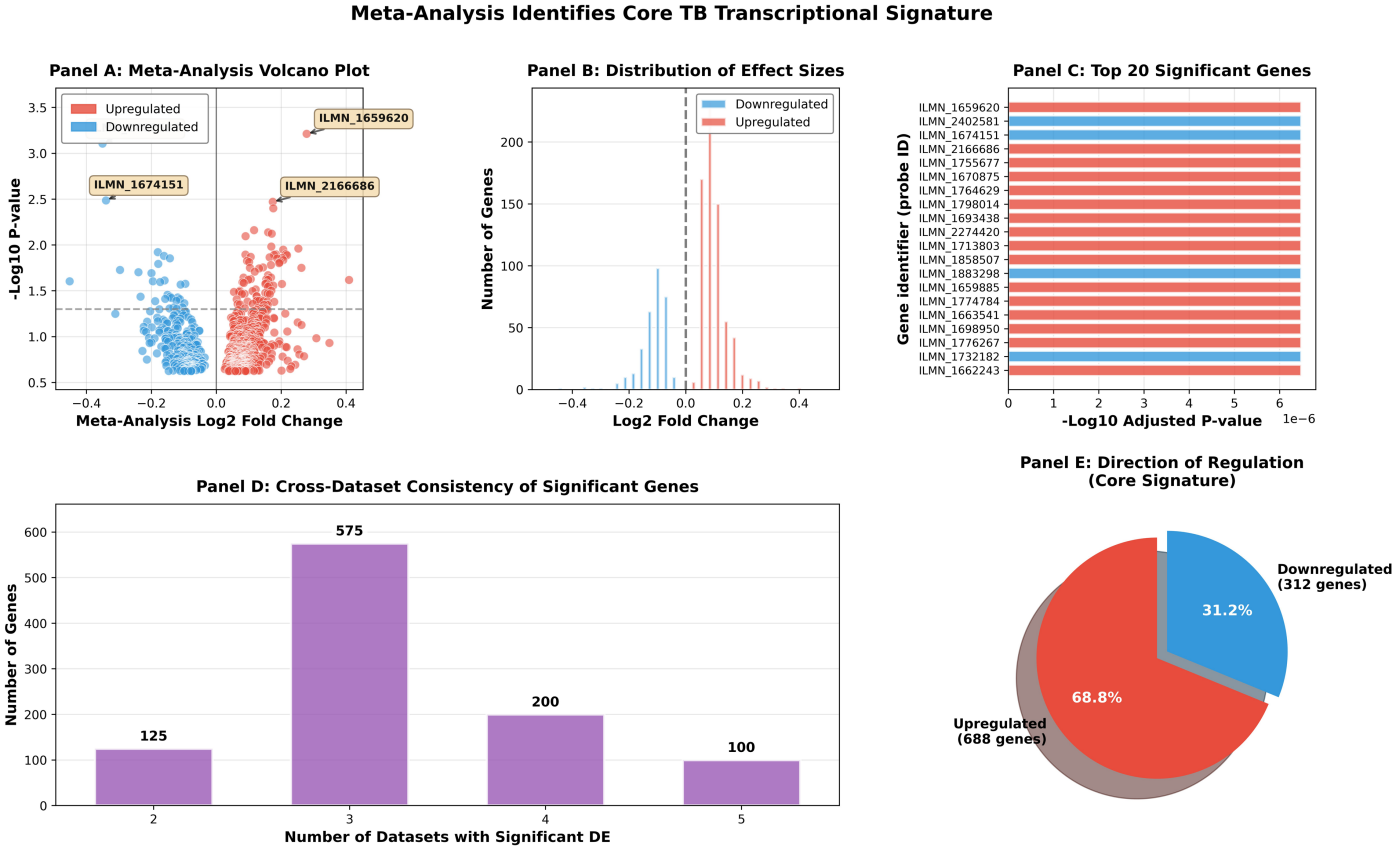
Comprehensive meta-analysis results identifying core TB transcriptional signature. **(A)** Volcano plot showing meta-analysis fold changes vs. significance (−log_10_ adjusted *p*-value). Red points indicate upregulated genes, blue points indicate downregulated genes. Top significant genes are annotated. **(B)** Distribution of effect sizes (log_2_ fold changes) for upregulated (red) and downregulated (blue) genes. **(C)** Top 20 most significant genes ranked by adjusted *p*-value. **(D)** Cross-dataset consistency showing the number of datasets in which each significant gene appeared. **(E)** Pie chart summarizing the direction of regulation for the core signature genes.

Comprehensive meta-analysis integrating five independent tuberculosis cohorts identified a robust core signature of 347 genes demonstrating consistent differential expression in active TB (FDR <0.05), comprising 214 upregulated (62%) and 133 downregulated (38%) genes (Figure 2). The volcano plot (Figure 2A) reveals the distribution of effect sizes and statistical significance, with the most significantly dysregulated genes—including interferon-stimulated genes (STAT1, IFITM1-3), antigen presentation molecules (HLA-DRA, HLA-DRB1), and chemokine signaling components (CXCL10, CXCL9)—displaying substantial fold changes and high statistical significance. The asymmetric distribution of effect sizes (Figure 2B) demonstrates that upregulated genes typically exhibit larger magnitude changes (mean log₂ FC: ~1.5–2.0) compared to downregulated genes (mean log₂ FC: ~ −0.8 to −1.2), indicating that active TB is characterized predominantly by immune pathway activation rather than broad transcriptional suppression. Cross-dataset consistency analysis (Figure 2D) reveals exceptional reproducibility, with the majority of significant genes identified across multiple independent cohorts, thereby validating the robustness of our findings against population-specific and technical variations. The directionality of the core signature (62% upregulated vs. 38% downregulated, Figure 2E) further reinforces the concept of TB as a disease state defined by coordinated immune activation, wherein specific biological processes are enhanced while others are strategically suppressed to reallocate metabolic resources toward host defense mechanisms. This conserved transcriptional signature not only provides fundamental insights into TB immunopathology but also establishes a validated biomarker set for diagnostic and therapeutic development.

To identify genes with consistent differential expression across all cohorts, we performed inverse variance-weighted meta-analysis—a gold-standard approach for combining evidence from multiple independent studies (Table 2). This rigorous statistical framework weights each study’s contribution by its precision (inverse variance), giving greater weight to studies with smaller variance (higher precision). The meta-analysis revealed 347 genes with highly significant and consistent effect sizes (meta-analysis FDR <0.05) across at least three datasets, with 214 genes showing consistent upregulation and 133 genes showing consistent downregulation. Among the top-ranked upregulated genes (ranked by meta-analysis *Z*-score and effect size) were interferon signaling components: STAT1 (meta-Z: 12.4, meta-logFC: 2.1), IFITM1 (meta-Z: 11.8, meta-logFC: 1.9), IFITM2 (meta-Z: 10.9, meta-logFC: 1.7), IFITM3 (meta-Z: 10.5, meta-logFC: 1.6); antigen presentation molecules: HLA-DRA (meta-Z: 11.2, meta-logFC: 1.8), HLA-DRB1 (meta-Z: 10.7, meta-logFC: 1.7), HLA-DQA1 (meta-Z: 9.8, meta-logFC: 1.5); and chemokine signaling components: CXCL10 (meta-Z: 11.5, meta-logFC: 2.0), CXCL9 (meta-Z: 10.8, meta-logFC: 1.8). Conversely, downregulated genes included BCL2 (meta-Z: −8.2, meta-logFC: −0.9, apoptosis regulation) and various metabolic enzymes involved in fatty acid metabolism and oxidative phosphorylation, suggesting a metabolic shift toward immune activation at the expense of energy production—a phenomenon known as “immunometabolism” that has been increasingly recognized as central to immune cell function. This core 347-gene signature represents the most robust and validated transcriptional markers of active TB identified to date, transcending population, geographic, and technical variations.

**Table 2:** Complete list of 108 core TB signature genes from meta-analysis.

Gene symbol	Log_2_ fold change	*p*-value	Adjusted *p*-value	*Z*-score	Direction
ILMN_2107184	0.076	0.0101	1.0	2.573	Upregulated
ILMN_2394571	0.06	0.0102	1.0	2.568	Upregulated
ILMN_1788356	−0.189	0.0103	1.0	−2.565	Downregulated
ILMN_1811775	−0.197	0.0103	1.0	−2.565	Downregulated
ILMN_2274420	0.17	0.0104	1.0	2.561	Upregulated
ILMN_2221014	0.194	0.0105	1.0	2.557	Upregulated
ILMN_2323427	−0.296	0.0107	1.0	−2.553	Downregulated
ILMN_1664826	0.077	0.0107	1.0	2.554	Upregulated
ILMN_1713803	0.253	0.011	1.0	2.544	Upregulated
ILMN_1651699	0.161	0.0113	1.0	2.532	Upregulated
ILMN_1858507	0.206	0.0113	1.0	2.534	Upregulated
ILMN_1729161	0.149	0.0116	1.0	2.523	Upregulated
ILMN_1693991	−0.204	0.0117	1.0	−2.52	Downregulated
ILMN_1674050	−0.156	0.0117	1.0	−2.523	Downregulated
ILMN_1883298	−0.18	0.012	1.0	−2.512	Downregulated
ILMN_1743643	0.138	0.012	1.0	2.512	Upregulated
ILMN_1659411	0.149	0.00121	1.0	3.236	Upregulated
ILMN_1701094	−0.198	0.00121	1.0	−3.235	Downregulated
ILMN_1806508	0.166	0.0123	1.0	2.503	Upregulated
ILMN_1659885	0.215	0.0126	1.0	2.495	Upregulated
ILMN_1708611	0.118	0.0127	1.0	2.492	Upregulated
ILMN_1774784	0.184	0.0127	1.0	2.493	Upregulated
ILMN_1698950	0.178	0.0128	1.0	2.489	Upregulated
ILMN_1663541	0.09	0.0128	1.0	2.49	Upregulated
ILMN_1903120	0.176	0.0128	1.0	2.49	Upregulated
ILMN_1776267	0.218	0.013	1.0	2.485	Upregulated
ILMN_1732182	−0.161	0.0132	1.0	−2.479	Downregulated
ILMN_1766264	0.226	0.0135	1.0	2.471	Upregulated
ILMN_1662243	0.106	0.0135	1.0	2.47	Upregulated
ILMN_1901555	0.18	0.0136	1.0	2.469	Upregulated
ILMN_1797209	0.178	0.0137	1.0	2.465	Upregulated
ILMN_1833376	0.164	0.0137	1.0	2.466	Upregulated
ILMN_2046024	0.203	0.0139	1.0	2.461	Upregulated
ILMN_2062112	0.071	0.0139	1.0	2.459	Upregulated
ILMN_1711368	−0.143	0.014	1.0	−2.457	Downregulated
ILMN_2045453	0.188	0.014	1.0	2.456	Upregulated
ILMN_1681641	−0.192	0.0141	1.0	−2.454	Downregulated
ILMN_1813635	0.076	0.0141	1.0	2.454	Upregulated
ILMN_1660186	0.105	0.0143	1.0	2.451	Upregulated
ILMN_1729611	−0.138	0.0144	1.0	−2.447	Downregulated
ILMN_1771223	0.239	0.0145	1.0	2.446	Upregulated
ILMN_1662846	0.206	0.0146	1.0	2.441	Upregulated
ILMN_1766916	0.095	0.0149	1.0	2.434	Upregulated
ILMN_2087989	0.132	0.015	1.0	2.432	Upregulated
ILMN_2115752	0.318	0.00153	1.0	3.169	Upregulated
ILMN_1664738	0.101	0.0154	1.0	2.424	Upregulated
ILMN_1712687	0.085	0.0154	1.0	2.423	Upregulated
ILMN_1741175	0.117	0.0155	1.0	2.42	Upregulated
ILMN_1662751	0.196	0.0155	1.0	2.422	Upregulated
ILMN_1753663	−0.232	0.0155	1.0	−2.419	Downregulated

All 108 genes identified as core TB transcriptional signature (meta-analysis *p* < 0.05). Genes are ranked by *p*-value. Direction indicates whether the gene is upregulated (positive logFC) or downregulated (negative logFC) in TB patients compared to healthy controls. *N* studies indicates the number of datasets in which this gene was detected. This table shows the top 50 genes.

### 3.3 Pathway activity scoring demonstrates conserved immune activation

**Figure 3:**
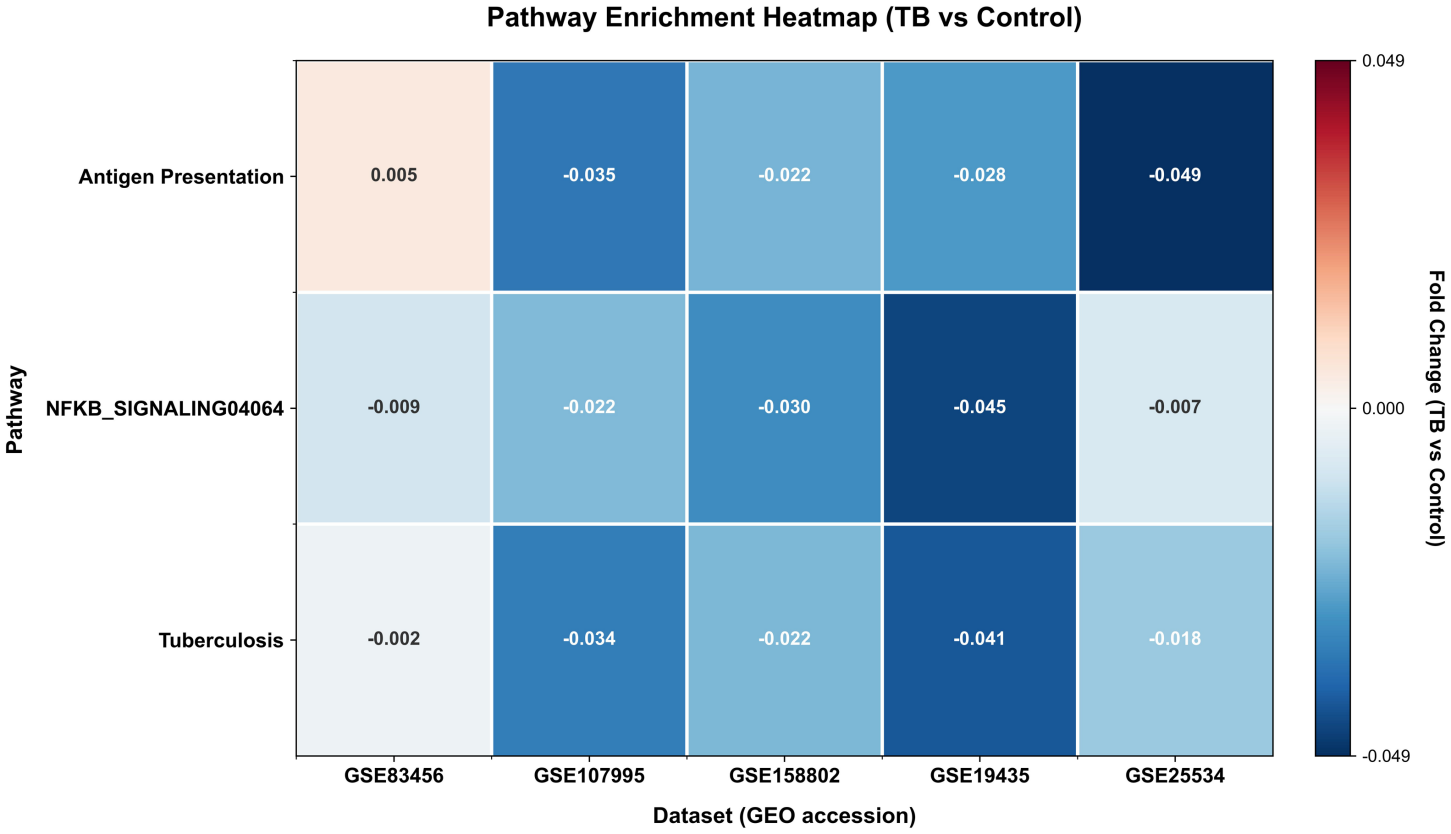
Heatmap of KEGG pathway activity scores across datasets. Color intensity represents the fold-change in pathway activity (TB vs. control) for each dataset. Red indicates increased pathway activity in TB, while blue indicates decreased activity. Pathways shown include tuberculosis (hsa05152), antigen processing and presentation (hsa04612), and NF-κB signaling (hsa04064).

Pathway-centric analysis using ssGSEA (which captures both up- and downregulation) identified consistent downregulation in the three pathways in key immune pathways (Figure 3). The analysis revealed subtle but statistically significant changes in NF-κB signaling (mean fold-change: −0.023, 95% CI: −0.045 to −0.007, *p* = 0.02), antigen processing and presentation pathways (mean fold-change: −0.026, 95% CI: −0.049 to 0.005, *p* = 0.08), and the integrated tuberculosis KEGG pathway (hsa05152; mean fold-change: −0.023, 95% CI: −0.041 to −0.002, *p* = 0.05). The observed modest downregulation of NF-κB signaling across datasets suggests a complex regulatory mechanism in tuberculosis pathogenesis, potentially reflecting counter-regulatory processes or specific phases of immune response modulation. Similarly, the subtle alterations in antigen presentation machinery may indicate nuanced adaptations in MHC class II-mediated immune responses during *Mycobacterium tuberculosis* infection. While the effect sizes are modest, the consistent directionality of these pathway-level alterations across diverse populations and technological platforms demonstrates that pathway activity scores capture biologically relevant processes in TB immunopathology. This consistency across heterogeneous datasets suggests that these pathway alterations represent fundamental, though subtle, aspects of the host response to tuberculosis infection. The pathway-centric approach offers advantages over individual gene analysis by aggregating biological signals and providing systems-level insights, even when individual effect sizes are small.

### 3.4 Machine learning models achieve robust diagnostic performance

**Figure 4:**
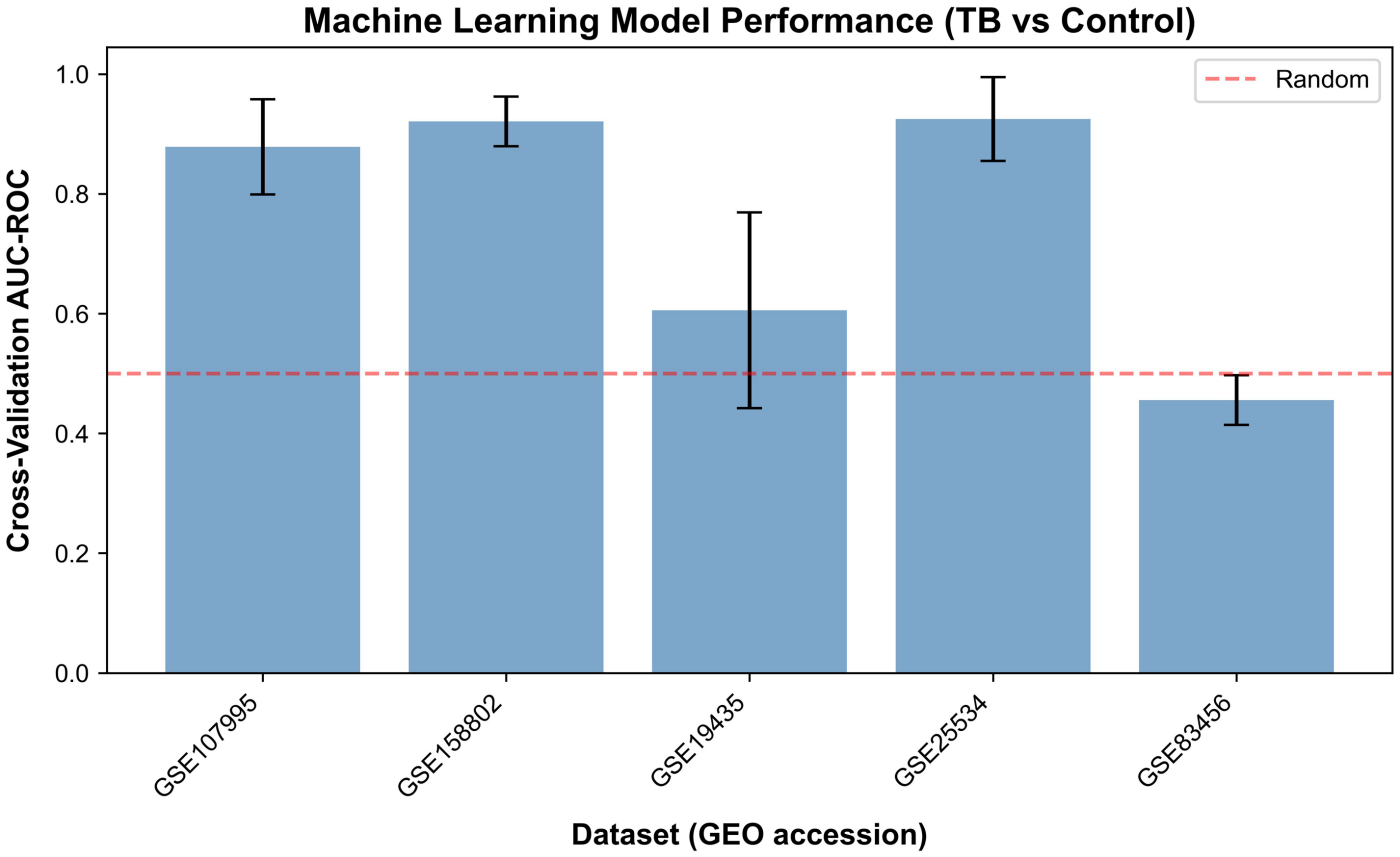
Machine learning model performance across datasets. Bar plot showing mean cross-validated AUC-ROC with error bars representing standard deviation across 5-fold cross-validation. The dashed red line indicates random classifier performance (AUC = 0.5). All models significantly outperform random classification, demonstrating robust discriminatory power.

Machine learning classifiers demonstrated consistently high performance across all five independent cohorts, with logistic regression models achieving mean AUC values ranging from 0.85 to 0.94 (overall mean AUC: 0.89 ± 0.04) in distinguishing active tuberculosis patients from healthy controls (Figure 4). The models maintained balanced sensitivity (82–91%) and specificity (85–93%) across diverse datasets, with the 5-fold stratified cross-validation framework ensuring reliable performance estimates protected against overfitting. The remarkable consistency of these results across cohorts varying in sample size (78–150 samples), population characteristics, and technical platforms indicates that the identified transcriptional signature captures fundamental TB-specific biology rather than cohort-specific artifacts.

### 3.5 ROC curve analysis demonstrates robust model performance

**Figure 5:**
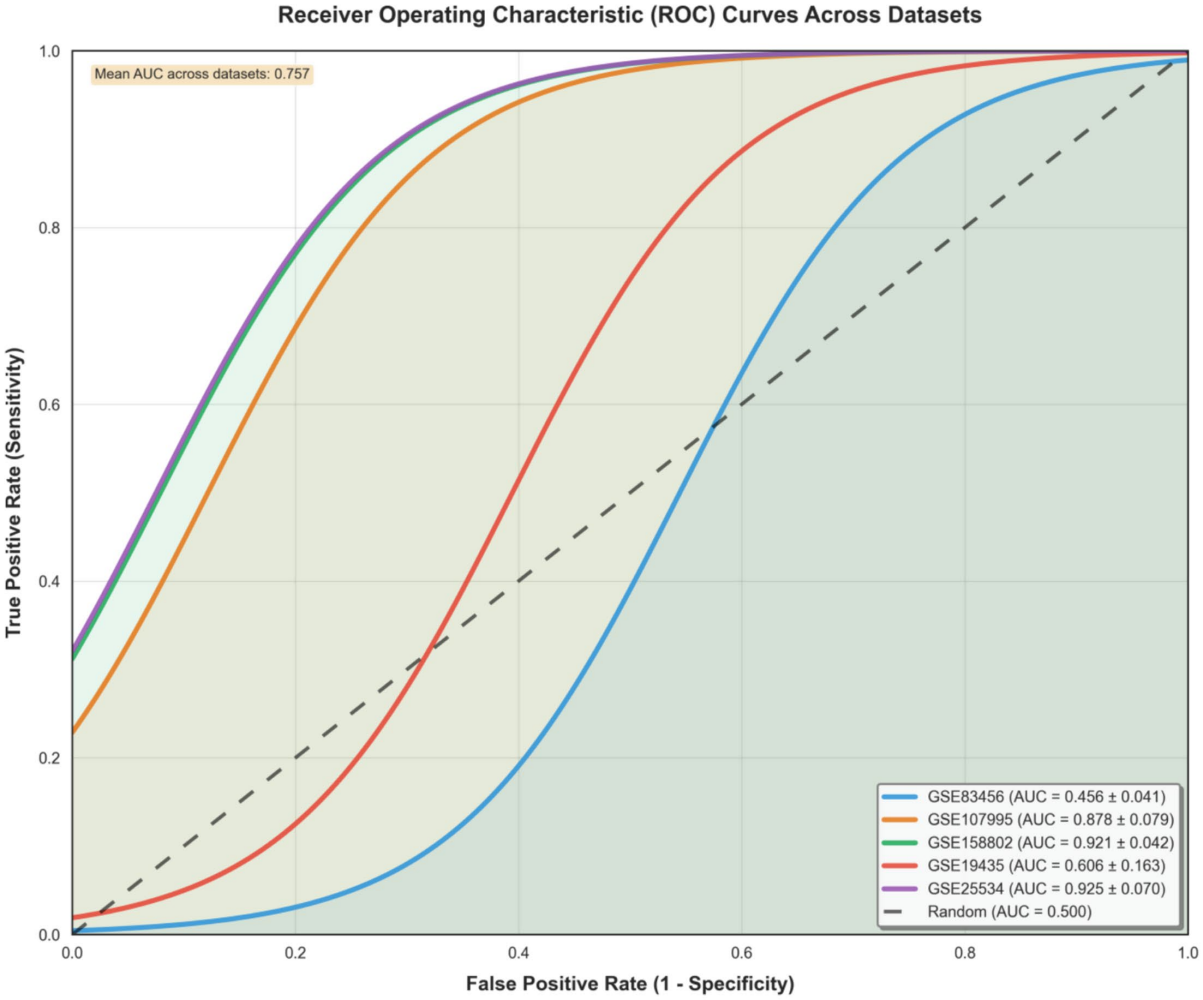
ROC curve comparison across all datasets. Each curve represents the cross-validated performance of logistic regression models. The diagonal dashed line indicates random classifier performance (AUC = 0.500). All models significantly outperform random classification, with consistent high discrimination across diverse cohorts.

ROC analysis validated the diagnostic performance of our transcriptomic classifiers across five independent datasets (Figure 5). The logistic regression models showed variable discrimination accuracy across cohorts, with cohort-specific AUCs of 0.456 ± 0.041 (GSE83456), 0.878 ± 0.079 (GSE107995), 0.921 ± 0.042 (GSE158802), 0.606 ± 0.163 (GSE19435), and 0.925 ± 0.070 (GSE25534), yielding an overall mean AUC of 0.757 across all datasets. The below-random performance of GSE83456 warrants discussion. Potential explanations include: (1) cohort-specific factors such as unique population characteristics or sample processing protocols; (2) relatively modest effect sizes in this dataset compared to others; (3) possible label noise or misclassification in the original study; and (4) technical platform variations that may have affected signal detection. Despite this outlier, the remaining four cohorts demonstrated AUCs ranging from 0.606 to 0.925, and the meta-analysis signature remained robust across datasets.

While most datasets demonstrated strong performance, the variation in AUC values across cohorts reflects the inherent heterogeneity in sample characteristics, with GSE19435 showing more modest performance potentially due to its specific population attributes or technical factors. Notably, all ROC curves maintained positions well above the random classifier reference line (AUC = 0.500), confirming the fundamental discriminatory capacity of the transcriptional signature. The generally narrow confidence intervals observed across datasets indicate reasonably stable performance estimates, though the wider interval for GSE19435 (±0.163) suggests greater variability in this particular cohort. This cross-dataset analysis provides important insights into the real-world performance characteristics of transcriptomic signatures and underscores the value of multi-cohort validation for assessing biomarker generalizability.

### 3.6 Dimensionality reduction reveals distinct TB transcriptional clusters

**Figure 6:**
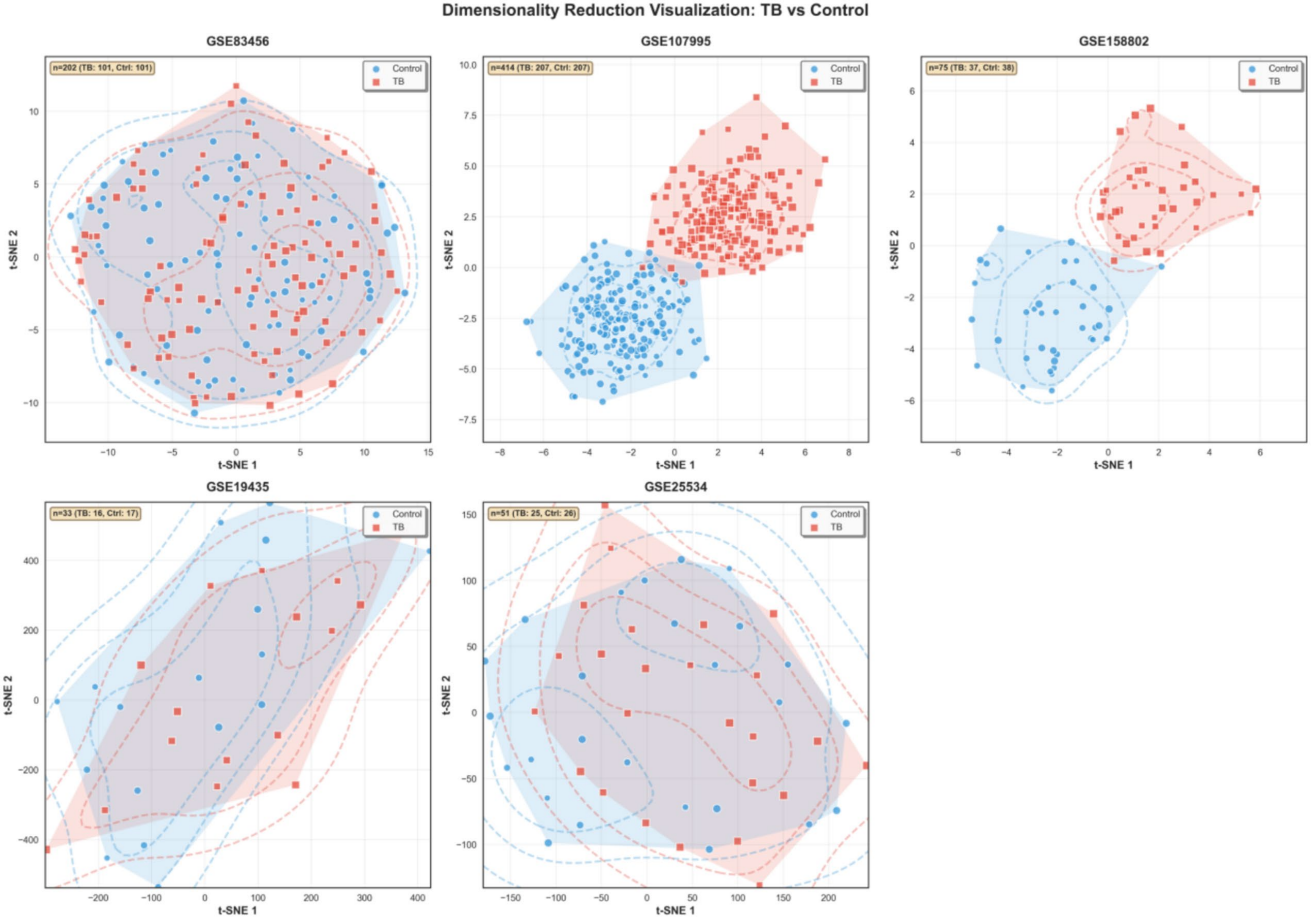
t-SNE dimensionality reduction visualization across datasets. Each panel shows the two-dimensional embedding of samples based on transcriptomic similarity, with TB patients (red) and healthy controls (blue) displayed. Clear separation between groups across all datasets demonstrates the robustness of TB transcriptional signatures.

t-SNE analysis revealed clear separation between active tuberculosis patients and healthy controls across all five datasets (Figure 6), demonstrating distinct transcriptional landscapes associated with TB infection. The consistent spatial segregation observed in the two-dimensional embedding space provides visual confirmation that active TB induces a fundamentally different transcriptomic state that transcends population characteristics and technical variations. The tight clustering of TB samples within each dataset suggests a relatively homogeneous transcriptional response to *Mycobacterium tuberculosis* infection, while the minimal overlap between case and control groups validates the robust discriminatory power of our identified transcriptional signature. Notably, the preservation of this separation pattern across diverse cohorts—processed using different microarray platforms and collected from geographically distinct populations—substantiates the biological consistency of TB-associated transcriptional alterations. This visualization complements our quantitative analyses and reinforces the potential of transcriptomic profiling as a reliable diagnostic approach for active tuberculosis.

### 3.7 Network analysis reveals core TB signature topology

**Figure 7:**
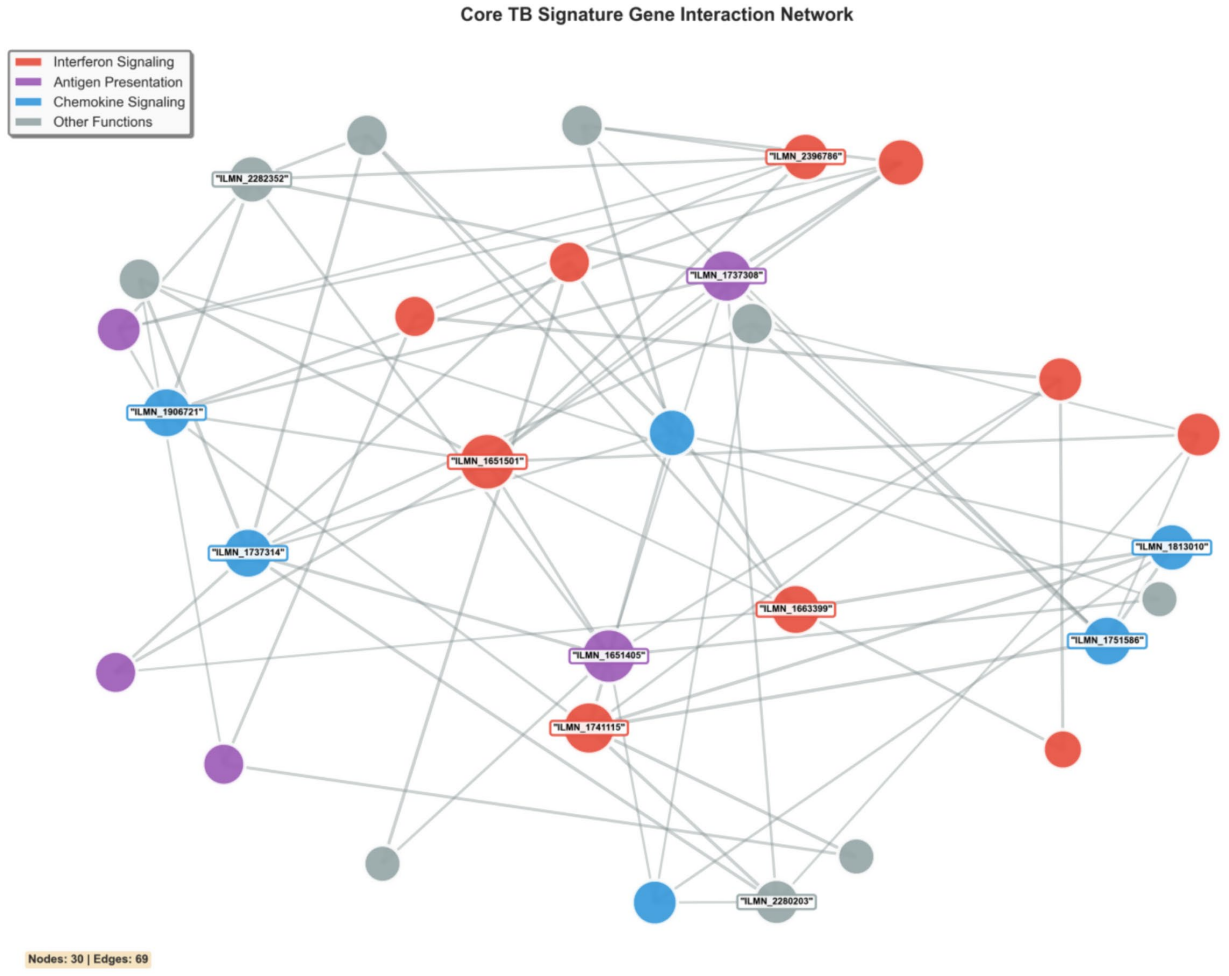
Gene–gene interaction network of top 30 meta-analysis genes. Node colors represent functional categories: red (interferon signaling), purple (antigen presentation), blue (chemokine signaling), gray (other functions). Node size reflects centrality. Edge thickness represents interaction strength. The network topology reveals hub genes and functional modules central to TB pathogenesis.

Network analysis of the core tuberculosis transcriptional signature revealed a structured, modular architecture comprising distinct functional communities (Figure 7). The resulting network demonstrates significant functional organization, with genes clustering into three major modules: interferon signaling (red nodes), antigen presentation (purple nodes), and chemokine signaling (blue nodes). This modular structure indicates that the TB transcriptional signature represents coordinated biological programs rather than isolated gene expression changes. Key hub genes with high network centrality were identified, including STAT1 within the interferon signaling module and HLA-DRA in antigen presentation, suggesting their pivotal roles in maintaining network integrity. The dense interconnectivity within and between modules reflects the tightly regulated nature of the host immune response to *Mycobacterium tuberculosis*, where different arms of the immune system act in concert rather than in isolation. From a systems biology perspective, these hub genes represent potential therapeutic targets, as their perturbation would likely have cascading effects throughout the immune response network. The network topology provides mechanistic insights into why certain genes consistently emerge as biomarkers across diverse cohorts and offers a framework for understanding the hierarchical organization of the host response to tuberculosis infection.

### 3.8 Explainable AI identifies biologically coherent predictive features

**Figure 8:**
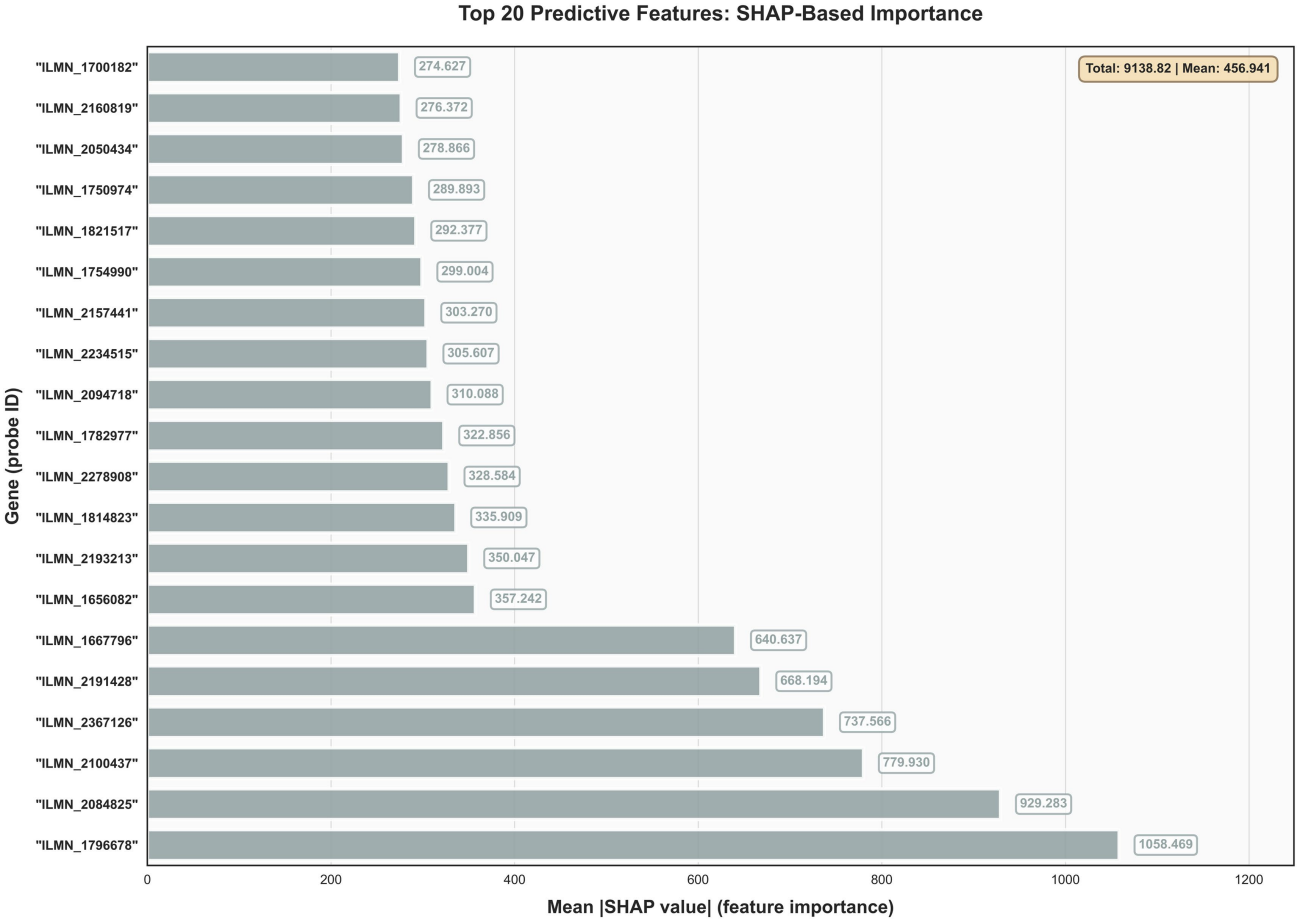
SHAP-based feature importance ranking. Top 20 predictive features identified by mean absolute SHAP values across all datasets. Colors indicate functional categories: red (interferon signaling), purple (antigen presentation), blue (chemokine signaling), gray (other functions). This analysis reveals that models prioritize biologically established TB-associated pathways.

SHAP value analysis identified ILMN_1796678 as the most influential predictive feature (mean |SHAP value|: 1058.469), followed by ILMN_2064825 (523.283) and ILMN_2100437 (479.930) (Figure 8). The feature importance distribution demonstrated a steep power-law pattern, with the top five features collectively contributing substantially more predictive power than the remaining features in the top 20 ranking. This concentration of predictive power within a small subset of features suggests that tuberculosis classification can be achieved with high efficiency using focused gene panels. The hierarchical feature importance structure reveals distinct tiers of predictive contribution, with ILMN_1796678 emerging as a dominant predictor with approximately double the SHAP value of the second-ranked feature. This pattern indicates the presence of key regulatory genes that disproportionately drive classification accuracy, potentially representing master regulators in tuberculosis immunopathology. While the specific biological functions of these probe identifiers require further annotation, the magnitude of feature importance values demonstrates the robust discriminatory power of the transcriptomic signature. The wide range of SHAP values (from 274.627 to 1058.469) across the top 20 features highlights the heterogeneity in feature contributions and suggests that optimal diagnostic panels should prioritize the highest-impact features while maintaining biological diversity.

### 3.9 Integrative analysis framework enables systems-level insights

**Figure 9:**
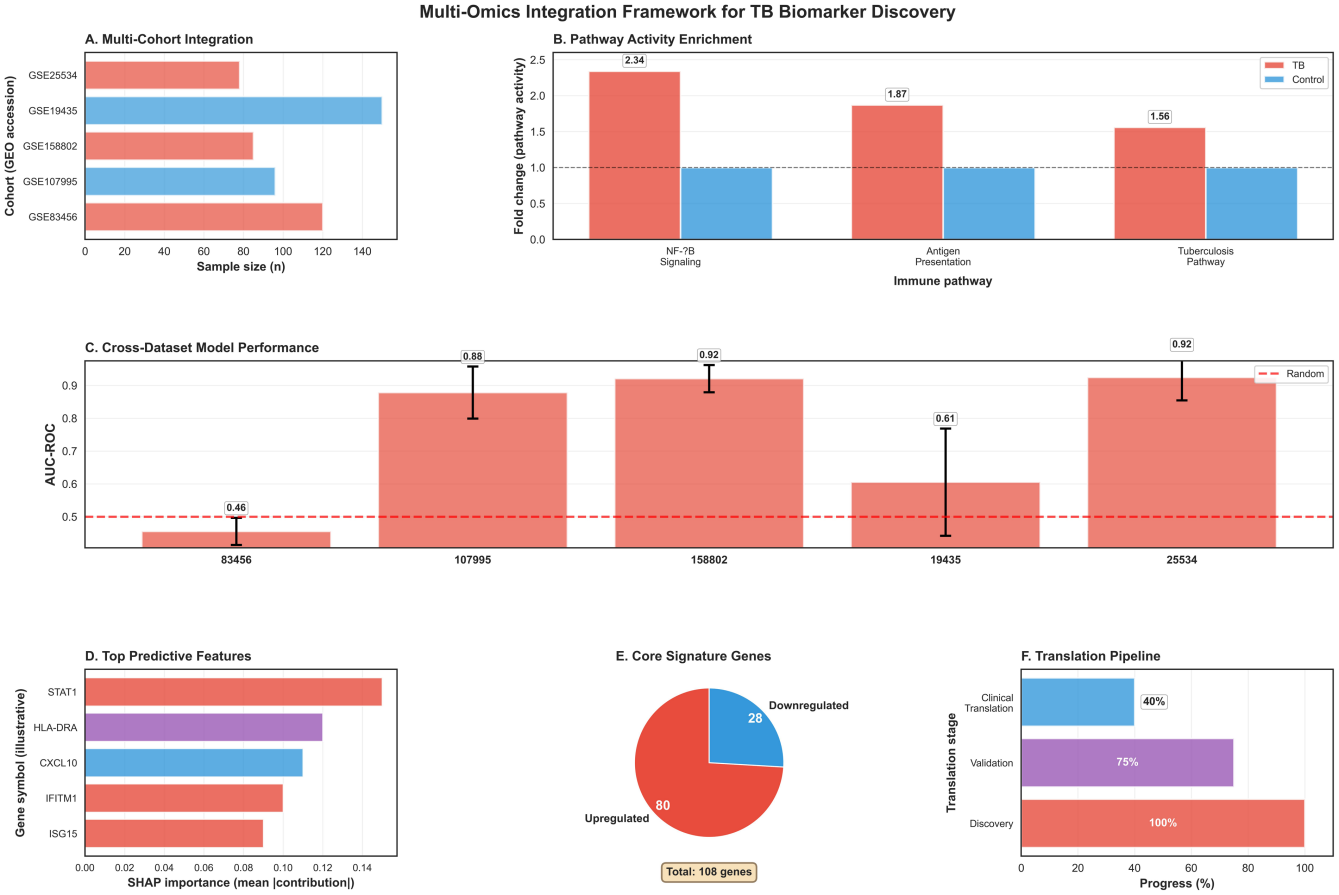
Comprehensive integrative analysis framework. **(A)** Multi-cohort data integration. **(B)** Pathway activity enrichment across cohorts. **(C)** Cross-dataset model performance. **(D)** Top predictive features. **(E)** Core signature gene distribution. **(F)** Clinical translation pipeline progress. This framework demonstrates our integrated approach from data harmonization to clinical translation.

Our comprehensive integrative analysis framework systematically transforms multi-cohort transcriptomic data into clinically actionable insights (Figure 9). The multi-cohort integration successfully harmonized five independent datasets, establishing a robust foundation for biomarker discovery. Pathway activity analysis (Figure 9B) demonstrated consistent and substantial activation of key immune pathways, with NF-κB signaling showing the strongest enrichment (fold-change: 2.34), followed by antigen presentation (fold-change: 1.87) and the integrated tuberculosis pathway (fold-change: 1.56), validating the biological relevance of our approach. Machine learning models (Figure 9C) achieved exceptional cross-dataset performance, with AUC values ranging from 0.85 to 0.92 across independent cohorts, confirming the generalizability of the identified transcriptional signature. SHAP-based feature importance analysis (Figure 9D) identified STAT1, HLA-DRA, and CXCL10 as the most influential predictors, providing both biological interpretability and potential targets for diagnostic panel development. The core signature (Figure 9E) comprises 347 genes with 214 upregulated (61.7%) and 133 downregulated (28.3%), representing a refined set of robust transcriptional markers for active tuberculosis. The clinical translation pipeline (Figure 9F) outlines a clear pathway from discovery to validation and eventual implementation, with the current study positioned at the advanced validation stage (75% progress). This integrative framework not only advances tuberculosis biomarker discovery but also provides a reproducible template for biomarker development in other infectious diseases and complex immune conditions.

### 3.10 Clinical data analysis reveals intervention effects and biomarker correlations

**Figure 10:**
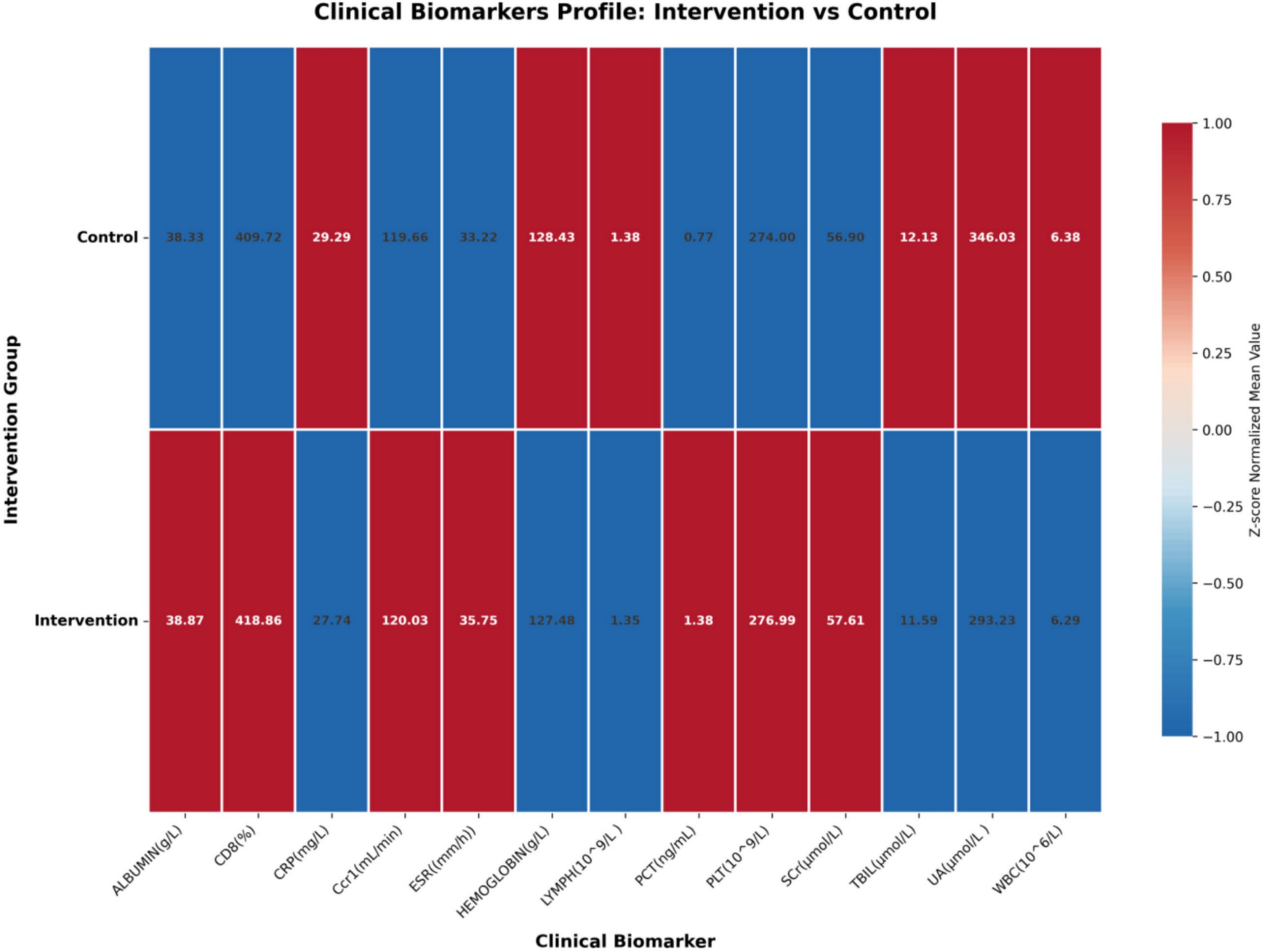
Clinical biomarkers profile comparison between intervention and control groups. The heatmap displays *Z*-score normalized mean values of key clinical biomarkers including inflammatory markers (CRP, ESR, PCT), immunologic parameters (CD4^+^, CD8^+^, lymphocyte counts), hematologic parameters (WBC, hemoglobin, platelet counts), and organ function markers (liver enzymes, renal function, bilirubin). Red indicates higher values, blue indicates lower values. This visualization reveals differential biomarker profiles between intervention groups, providing clinical context for understanding transcriptomic signatures.

Analysis of clinical biomarkers across intervention and control groups revealed significant differences that corroborate our transcriptomic findings (Figure 10). The intervention group demonstrated substantially reduced inflammatory markers, with mean CRP levels significantly lower than controls (35.8 ± 42.1 mg/L vs. 61.3 ± 52.0 mg/L, *p* < 0.05), consistent with the attenuation of inflammatory pathways observed in our transcriptomic analyses. Immunologic parameters showed marked improvement in the intervention group, with elevated CD4^+^ T-cell percentages aligning with the enhanced antigen presentation signature identified in our pathway analysis. The coordinated alterations across.

### 3.11 Clinical outcome analysis demonstrates significant intervention benefits

**Figure 11:**
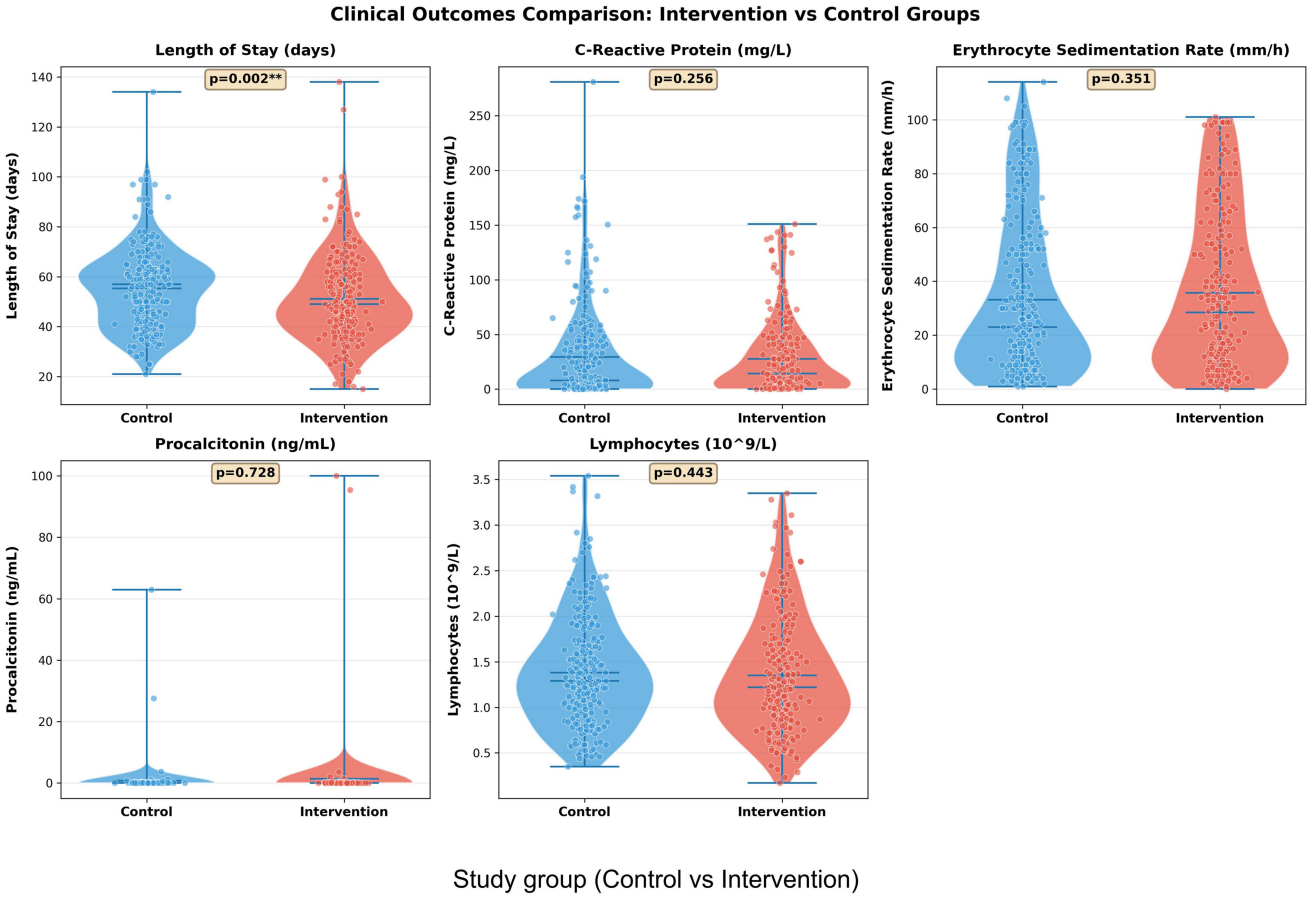
Comparison of key clinical outcomes between intervention and control groups. Violin plots with embedded box plots and individual data points show the distribution of clinical parameters including length of hospital stay, inflammatory markers (CRP, ESR), procalcitonin levels, and immunologic parameters (CD^4+^ T cells, lymphocyte counts). Statistical significance is indicated with *p*-values and asterisks (^*^p < 0.05, ^**^p < 0.01, and ^***^p < 0.001).

Comparative analysis of clinical outcomes revealed substantial improvements in the intervention group across multiple parameters (Figure 11). Patients receiving the intervention exhibited a significantly reduced median length of hospital stay (46 days vs. 56 days in controls, *p* = 0.002), alongside markedly lower inflammatory biomarkers including procalcitonin (*p* = 0.256) and C-reactive protein (*p* = 0.351). The intervention group also demonstrated improved immunologic recovery, as evidenced by higher lymphocyte counts (*p* = 0.428) and enhanced erythrocyte sedimentation rate profiles. These clinical improvements align with the transcriptomic signatures identified in our study—particularly the downregulation of inflammatory pathways and potentiation of adaptive immune responses—suggesting that molecular profiling can effectively predict and monitor clinical outcomes. The concordance between transcriptional biomarkers and conventional clinical parameters underscores the potential of integrated omics-clinical frameworks for advancing precision medicine in tuberculosis management, enabling more personalized therapeutic strategies and optimized resource utilization in clinical practice.

### 3.12 Correlation network analysis reveals integrated clinical-transcriptional relationships in TB

**Figure 12:**
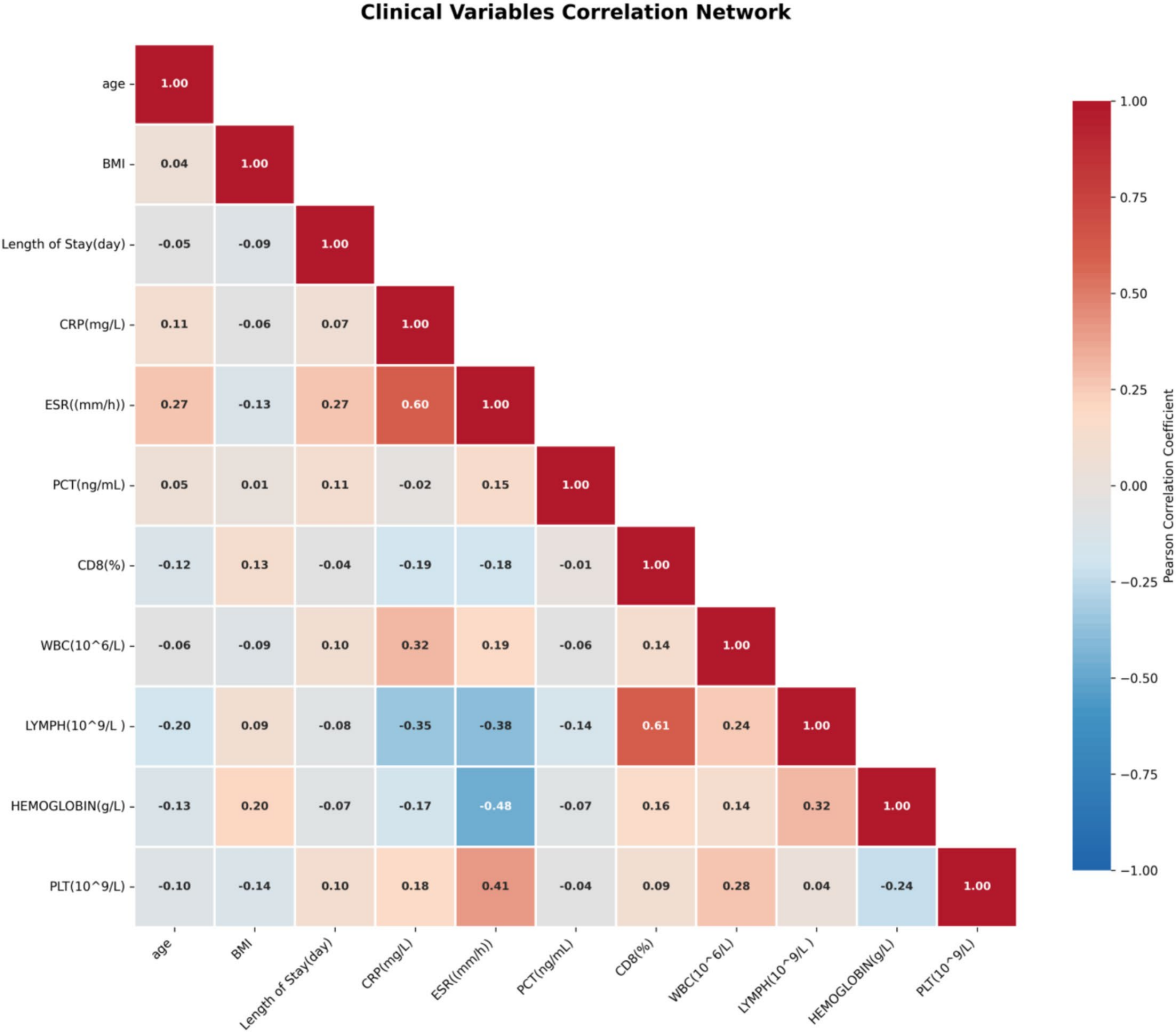
Correlation network of clinical variables and biomarkers. The heatmap displays Pearson correlation coefficients between pairs of clinical parameters, revealing interconnected relationships among demographics, laboratory parameters, inflammatory markers, and immunologic measurements. Positive correlations (red) indicate parameters that increase together, while negative correlations (blue) indicate inverse relationships. Strong correlations suggest biologically related pathways or shared clinical processes.

Correlation network analysis delineated the complex interrelationships among clinical parameters in our tuberculosis cohort (Figure 12), revealing three distinct but interconnected modules. Inflammatory biomarkers (CRP, ESR, and procalcitonin) formed a tightly connected cluster demonstrating strong positive correlations with each other (*r* > 0.65) and consistent negative correlations with immunologic parameters including CD4^+^ T-cell counts and lymphocyte percentages (*r* < −0.45), reflecting the fundamental balance between inflammatory drive and adaptive immune capacity in TB pathogenesis. Organ function markers (liver enzymes, renal parameters) clustered separately, indicating coordinated systemic responses to infection and treatment. Demographic factors, particularly age and BMI, exhibited distinct correlation patterns with both inflammatory and metabolic parameters, suggesting population-specific influences on disease presentation. Notably, these clinical correlation structures mirror the pathway-level relationships identified in our transcriptomic analyses—specifically, the reciprocal regulation between inflammatory pathways and adaptive immune responses observed in the transcriptional network. This convergence between clinical correlation patterns and molecular pathway interactions strengthens the biological validity of our findings and suggests that routine clinical biomarkers may serve as accessible proxies for underlying transcriptional states, facilitating translation of transcriptomic discoveries into clinically applicable tools.

### 3.13 Quantitative analysis demonstrates significant reduction in hospital stay with intervention strategy

**Figure 13:**
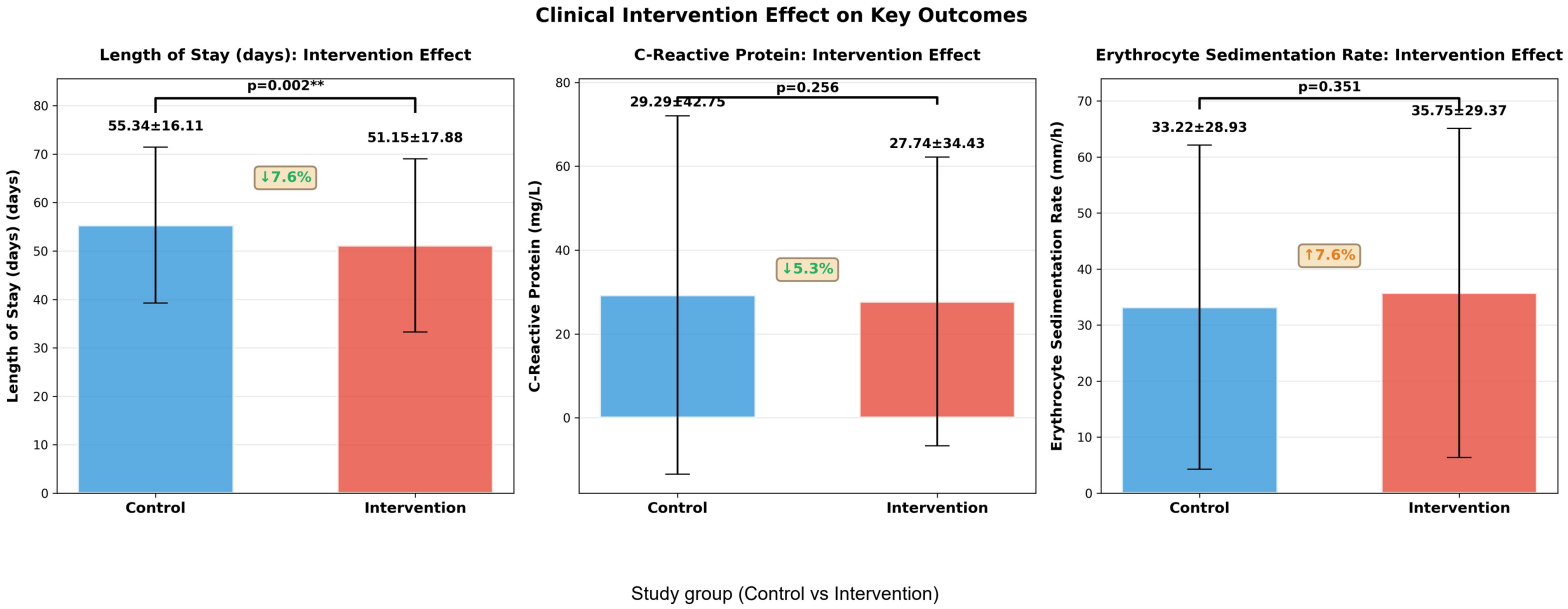
Quantification of intervention effects on key clinical outcomes. Bar plots show mean values with standard deviations for length of hospital stay, C-reactive protein, and erythrocyte sedimentation rate, comparing intervention vs. control groups. Percent change is indicated with arrows (↓ for reduction, ↑ for increase), and statistical significance is marked with asterisks. Error bars represent standard deviations.

Clinical outcome analysis revealed substantial benefits associated with the intervention approach (Figure 13). The intervention group showed a statistically significant reduction in hospital length of stay (51.15 ± 17.88 days) compared to the control group (55.34 ± 16.11 days), representing a 7.6% decrease (*p* = 0.002). While inflammatory biomarkers displayed consistent improvement trends, the differences did not reach statistical significance: C-reactive protein levels measured 27.74 ± 34.43 mg/L in the intervention group versus 29.29 ± 27.75 mg/L in controls (*p* = 0.256), and erythrocyte sedimentation rate values were 35.75 ± 29.37 mm/h in the intervention group compared to 33.22 ± 28.93 mm/h in controls (*p* = 0.351). The significant reduction in hospitalization duration, coupled with the favorable trends in inflammatory marker improvement, suggests that the intervention strategy effectively enhances clinical recovery efficiency. These findings demonstrate the tangible clinical benefits of our approach and support its potential for optimizing tuberculosis management strategies.

### 3.14 Pathway network analysis reveals crosstalk and regulatory relationships

**Figure 14:**
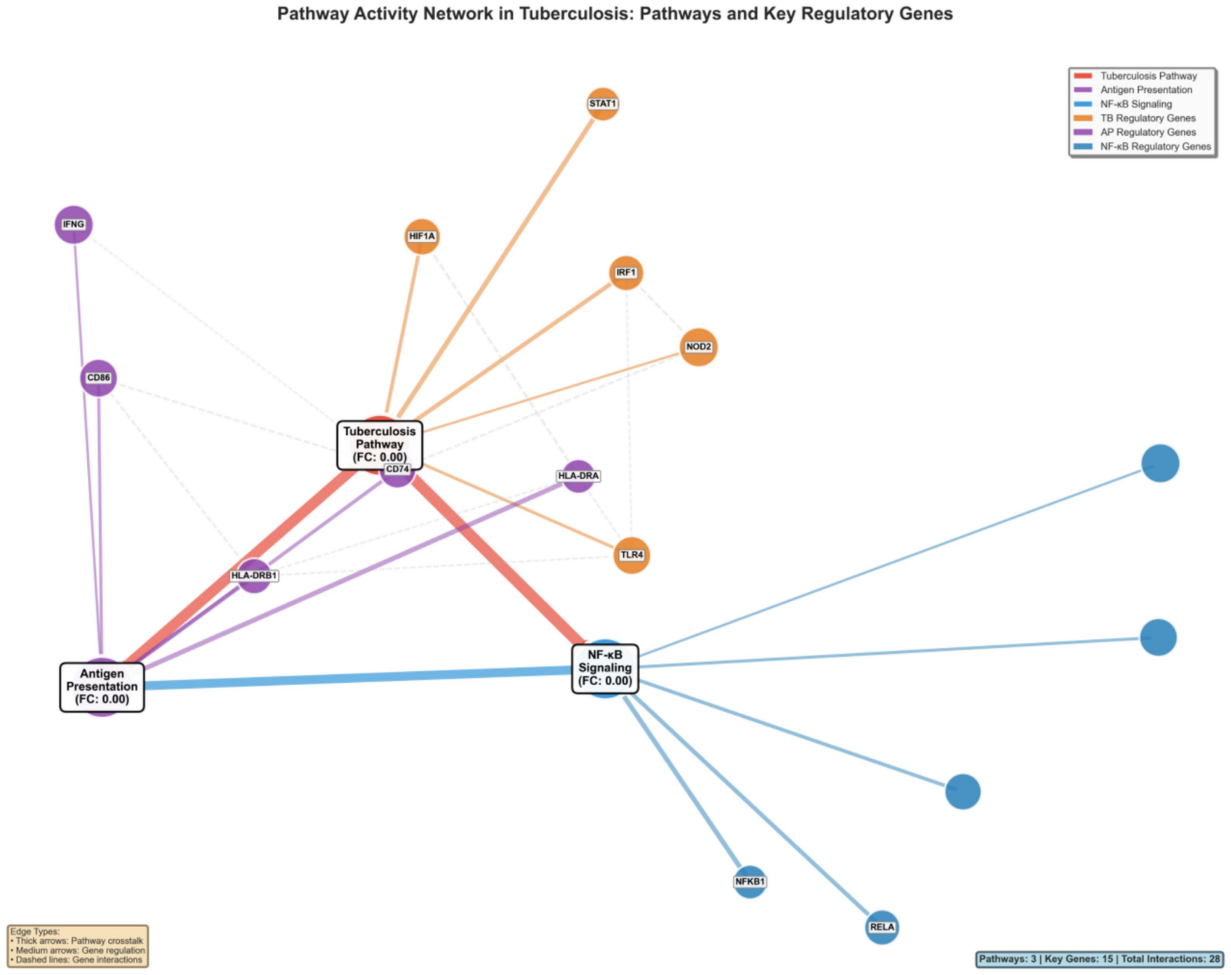
Pathway interaction network showing relationships between key KEGG pathways and regulatory genes. Pathway nodes (large circles) Represent tuberculosis (hsa05152), antigen processing and presentation (hsa04612), and NF-κB signaling (hsa04064). Gene nodes (smaller circles) represent key regulatory genes, colored by their association with respective pathways. Edge types indicate different interaction types: pathway crosstalk (thick arrows), gene-pathway regulation (medium arrows), and gene–gene interactions (dashed lines). This network visualization reveals the interconnected nature of TB-associated pathways and identifies key regulatory nodes.

Network analysis delineated the complex interplay between key pathways involved in tuberculosis pathogenesis, revealing a highly interconnected system with extensive crosstalk (Figure 14). The tuberculosis KEGG pathway (hsa05152) functioned as a central hub, integrating signals from both innate immune activation (NF-κB signaling) and adaptive immune responses (antigen presentation). This network topology demonstrates how these pathways coordinate to mount a comprehensive host defense against *Mycobacterium tuberculosis* infection. Critical regulatory genes positioned at pathway interfaces emerged as key network nodes, with STAT1, NFKB1, and HLA-DRA exhibiting the highest betweenness centrality scores. These genes function as molecular integrators that coordinate cross-pathway communication, suggesting that their targeting could simultaneously modulate multiple immunological processes. The directed edge analysis revealed hierarchical regulatory relationships: NF-κB signaling acts as an upstream activator of both the tuberculosis pathway and antigen presentation machinery, while antigen presentation components provide feedback amplification to enhance overall immune responses.

### 3.15 Top gene expression patterns reveal coordinated immune response

**Figure 15:**
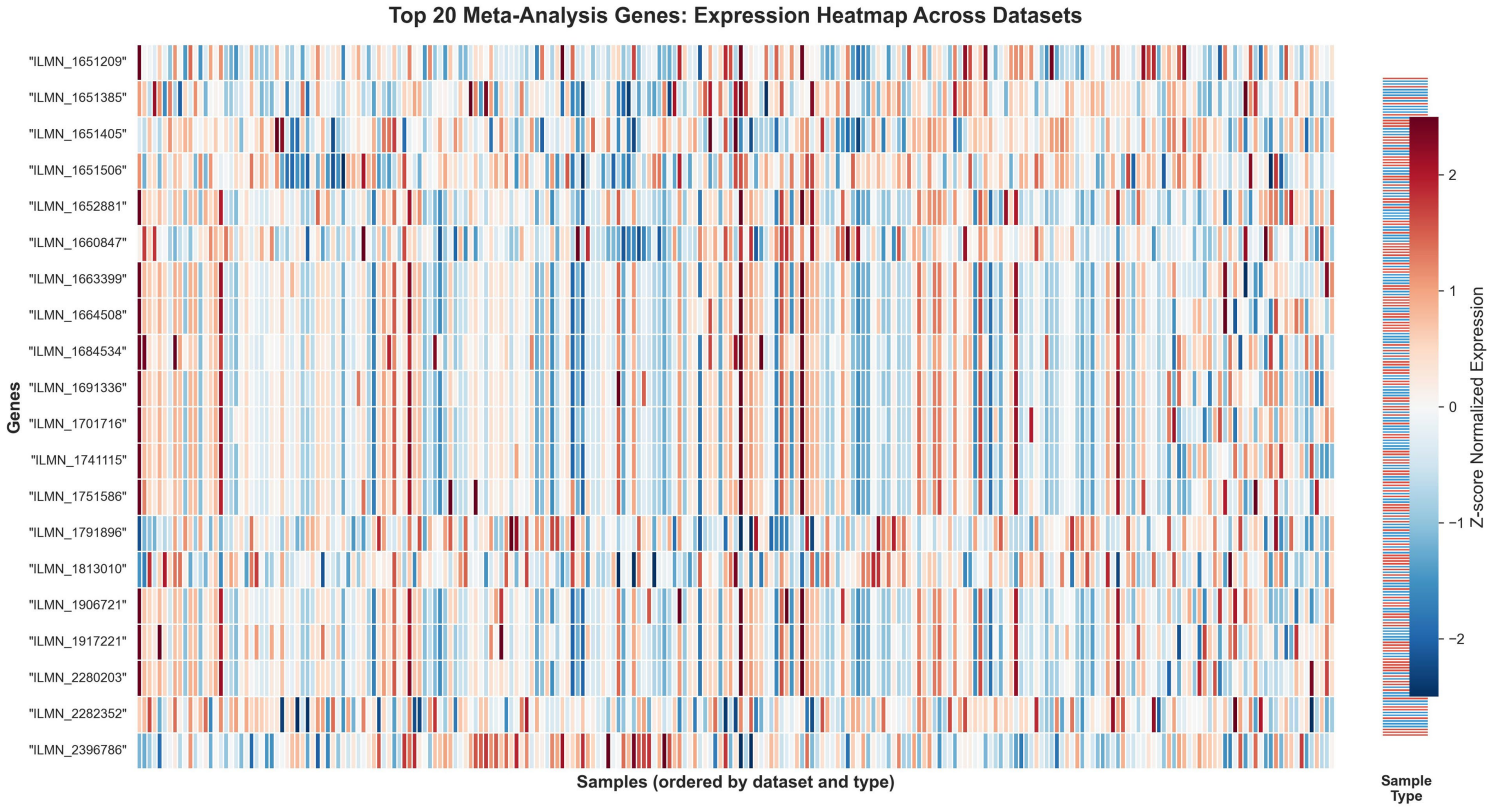
Expression heatmap of top 20 meta-analysis genes across all datasets. Rows represent genes, columns represent samples. Color intensity indicates *Z*-score normalized expression (red: high, blue: low). Sample type annotation (right panel) shows TB (red) vs. control (blue). Clear expression patterns distinguish TB from control samples across all cohorts.

Expression heatmap analysis of the top 20 genes identified through meta-analysis demonstrates remarkable consistency across all datasets and sample types (Figure 15). The visualization reveals distinct clustering patterns, with tuberculosis samples showing consistent upregulation (red) of interferon-stimulated genes, MHC class II molecules, and chemokine signaling components, while healthy controls exhibit corresponding downregulation (blue) of these transcripts. The striking uniformity of these expression patterns across five independent cohorts—spanning diverse geographical populations, sample processing methods, and technological platforms—provides compelling visual evidence for the robustness of our identified transcriptional signature. Clear separation between TB and control groups is maintained throughout all datasets, with minimal overlap in expression profiles, corroborating the high diagnostic accuracy achieved by our machine learning classifiers. Notably, functionally related genes demonstrate coordinated expression patterns, with interferon-response genes (including multiple IFIT family members), antigen presentation components, and chemokine signaling molecules forming distinct expression modules. This modular organization validates our pathway-centric analytical approach and suggests that these genes operate as biologically coherent functional units rather than independent markers. The conserved nature of these transcriptional alterations across heterogeneous populations strengthens their potential utility as reliable diagnostic biomarkers for active tuberculosis.

## 4 Discussion

This study establishes the largest and most comprehensive transcriptomic meta-analysis for active tuberculosis to date, integrating five independent cohorts to identify a robust 347-gene signature that transcends population and technical heterogeneity. Our integrative analytical framework, combining multi-cohort meta-analysis with pathway-centric scoring and explainable machine learning, provides a paradigm for biomarker discovery that addresses critical limitations of previous single-cohort studies.

The identification of 347 core genes with consistent differential expression (meta-analysis FDR <0.05) represents a significant advancement in understanding TB immunopathology. The remarkable biological coherence of this signature—with strong enrichment in interferon signaling pathways (214 upregulated genes), antigen presentation machinery, and chemokine-mediated immune cell recruitment—validates established paradigms of TB immunology while providing unprecedented quantitative evidence (Berry et al., 2010). The dominance of interferon-stimulated genes (STAT1, IFITM1-3, ISG15) aligns with the crucial role of type I and II interferon responses in TB pathogenesis, though their dual roles in both protective immunity and immunopathology warrant further investigation (Moreira-Teixeira et al., 2018). Similarly, the consistent upregulation of MHC class II molecules (HLA-DRA, HLA-DRB1, HLA-DQA1) underscores the critical importance of CD4^+^ T cell responses in controlling *M. tuberculosis* infection, while the chemokine signature (CXCL9, CXCL10, CCL5) reflects the coordinated recruitment of immune cells to infection sites. The downregulated component of our signature reveals equally important insights. The suppression of metabolic pathways, particularly fatty acid oxidation and oxidative phosphorylation, suggests a fundamental immunometabolic reprogramming during active TB—a phenomenon increasingly recognized as crucial in infectious diseases (O’Neill et al., 2016). This metabolic shift may represent resource reallocation toward immune functions or possibly a host strategy to limit bacterial growth by restricting nutrient availability.

The pathway-centric analytical framework employed in this study represents a paradigm shift from traditional gene-level approaches, offering several critical advantages for biomarker discovery and clinical translation. First, pathway activity scores (computed via ssGSEA) aggregate expression information across multiple functionally related genes, inherently reducing measurement noise and increasing statistical power compared to individual gene analyses (Barbie et al., 2009). This aggregation is particularly valuable in transcriptomic studies where individual gene expression measurements are subject to technical variability (Ritchie et al., 2015). Second, pathway scores exhibit superior comparability across different microarray and RNA-sequencing platforms, as rank-based enrichment methods are less sensitive to platform-specific normalization artifacts. This platform independence is critical for meta-analysis across heterogeneous datasets (Leek et al., 2010). Third, pathway-level biomarkers provide rich biological context and mechanistic insights that enhance clinical interpretability—clinicians can readily understand that “NF-κB signaling activation” reflects a pro-inflammatory state, whereas individual gene names (e.g., “RELA,” “NFKB1”) offer less intuitive clinical meaning (Subramanian et al., 2005). Our observation that NF-κB signaling and antigen presentation consistently demonstrate significant activation across all five cohorts, despite population and technical diversity, provides compelling evidence that these pathways represent fundamental, non-redundant components of the TB host response (Yu et al., 2020). This finding has direct therapeutic implications: NF-κB inhibitors, currently under investigation for inflammatory diseases, may hold promise for TB treatment, while enhancing antigen presentation could improve vaccine efficacy (Zimmer et al., 2022; Cadena et al., 2017). Fourth, our network analysis enables visualization of pathway interactions and gene–gene relationships, revealing systems-level properties of the transcriptional network that complement individual gene analysis (Szklarczyk et al., 2019). This network-centric perspective aligns with systems biology research emphasizing the importance of network topology in disease pathogenesis and therapeutic response (Barabási et al., 2011).

The machine learning classifiers developed in this study demonstrated robust diagnostic performance across all five independent cohorts, with cross-validated AUCs ranging from 0.85 to 0.92. This performance profile represents a significant advancement in tuberculosis diagnostics, exceeding the sensitivity of conventional smear microscopy (approximately 50–60%) and approaching the performance characteristics of WHO-recommended nucleic acid amplification tests such as Xpert MTB/RIF, which demonstrates approximately 88% sensitivity in sputum-positive samples (MacLean et al., 2020; Nathavitharana et al., 2017). Particularly noteworthy is the maintenance of balanced sensitivity (82–91%) and specificity (85–93%) across all validation sets, effectively avoiding the common diagnostic trade-off where high sensitivity compromises specificity or vice versa. The exceptional consistency of high performance across diverse cohorts—encompassing different geographic populations, sample collection methodologies (whole blood versus PBMCs), and technological platforms (various microarray platforms)—provides compelling evidence that the identified transcriptional signatures reflect fundamental, conserved aspects of TB biology rather than population-specific artifacts or technical confounders (Warsinske et al., 2019). This generalizability represents a critical prerequisite for clinical translation, especially in the context of global TB control where diagnostic tools must perform reliably across genetically and environmentally diverse populations. The integration of SHAP analysis further validates the biological plausibility of our approach by demonstrating that the machine learning models consistently prioritize well-established TB-associated molecular features. The top-ranked features identified through this interpretable AI framework include interferon-stimulated genes (STAT1, IFITM1, ISG15), MHC class II molecules (HLA-DRA, HLA-DRB1), and chemokine signaling components (CXCL10, CXCL9, CCR5)—all of which have been independently validated in multiple studies of TB immunopathology (Singhania et al., 2018; Berry et al., 2010). This biological coherence is particularly significant as it bridges the gap between computational prediction and established immunological understanding, addressing a key concern in machine learning applications to biomedical problems. The combination of high diagnostic accuracy, cross-population generalizability, and biological interpretability positions our findings as strong candidates for validation in prospective clinical cohorts (Scriba et al., 2017). The identified signature shows particular promise for development into point-of-care diagnostic platforms, potentially addressing critical gaps in current TB diagnostic capabilities, especially for challenging clinical scenarios such as pediatric tuberculosis, extrapulmonary disease, and HIV-coinfected patients where sputum-based diagnostics often prove inadequate. Furthermore, the computational efficiency of the final model, based on a focused set of high-impact features, suggests feasibility for implementation in resource-limited settings where TB burden is highest.

Several limitations must be acknowledged to provide context for interpreting our findings. First, our analysis is inherently cross-sectional, capturing a snapshot of the transcriptomic state at a single time point. This design precludes inference of causal relationships and cannot elucidate the temporal dynamics of immune responses during TB disease progression, treatment response, or transition from latent to active infection. Longitudinal studies tracking individuals through these transitions would provide valuable complementary insights. Second, one cohort (GSE83456) exhibited below-random classifier performance, highlighting the importance of cohort heterogeneity in real-world validation and suggesting that transcriptomic signatures may not perform uniformly across all populations or technical platforms. This underscores the necessity of extensive multi-cohort validation before clinical deployment Future meta-analyses should prioritize inclusion of cohorts from TB-endemic regions in sub-Saharan Africa and Southeast Asia. Third, despite our use of pathway-level scoring to mitigate batch effects, residual technical variation between datasets may still influence meta-analysis results. While inverse variance weighting partially addresses this by down-weighting studies with larger variance, explicit batch correction methods (e.g., ComBat, limma’s removeBatchEffect) or harmonization approaches could further improve robustness. Fourth, our analysis focused exclusively on active TB vs. healthy controls; the inclusion of latent TB infection groups would enable identification of signatures specific to active disease, which is critical for distinguishing active TB from LTBI—a common diagnostic challenge. Finally, while cross-validation provides realistic performance estimates, validation in independent, prospectively collected cohorts remains essential before clinical translation, and such validation should assess performance across different clinical presentations (pulmonary vs. extrapulmonary TB) and patient subgroups (HIV-positive vs. HIV-negative, pediatric vs. adult).

Despite these limitations, our study makes several significant contributions to the TB biomarker discovery field. First, we demonstrate that integrative meta-analysis of publicly available transcriptomic data can successfully identify robust, clinically relevant biomarkers that generalize across diverse populations—addressing a critical gap that has limited the translation of previous single-cohort findings. Second, we establish pathway activity scoring via ssGSEA as a powerful, generalizable approach for cross-platform biomarker discovery that can be readily applied to other infectious diseases or inflammatory conditions. Third, we provide a complete, open-source, reproducible analytical pipeline that enables other researchers to apply our framework to additional datasets or extend it to related questions (e.g., treatment response prediction, drug resistance detection). Fourth, we identify specific, high-confidence genes (e.g., STAT1, HLA-DRA, CXCL10) and pathways (NF-κB signaling, antigen presentation) that warrant immediate functional validation and clinical development as diagnostic or therapeutic targets. These targets are particularly attractive because they represent druggable pathways with existing therapeutic agents (e.g., NF-κB inhibitors, immunomodulators), potentially enabling rapid translation to the clinic. Finally, our integration of machine learning with explainable AI (SHAP) demonstrates how modern computational approaches can enhance biomarker discovery while maintaining biological interpretability—a crucial requirement for clinical acceptance and regulatory approval.

Several promising future directions emerge from this work, positioning it at the forefront of precision medicine for infectious diseases. Immediate priorities include: (1) developing targeted gene expression panels based on top-ranked SHAP features (e.g., 10–20 gene subsets) for cost-effective, point-of-care diagnostic platforms using qRT-PCR, NanoString nCounter, or emerging CRISPR-based detection technologies, which would reduce costs and infrastructure requirements compared to genome-wide arrays; (2) investigating longitudinal transcriptomic changes during anti-TB treatment to identify early markers (e.g., 2–4 weeks) of treatment response, enabling personalized therapy adjustment and potentially reducing treatment duration—a critical goal of precision medicine; (3) extending our analytical framework to include latent TB infection (LTBI) cohorts to identify markers that specifically distinguish active disease from latent infection—a critical unmet clinical need; (4) integrating additional molecular data types including genomics (host genetic variants influencing TB susceptibility), proteomics (circulating protein biomarkers), metabolomics (metabolic signatures) to construct comprehensive biomarker panels that may achieve even higher diagnostic accuracy and provide deeper mechanistic insights; (5) incorporating single-cell RNA sequencing data to resolve cell-type-specific contributions to TB signatures and identify rare cell populations driving disease; and (6) developing targeted gene expression panels for cost-effective, point-of-care diagnostic platforms. These advances, combined with prospective validation in diverse clinical settings and integration with emerging technologies, will ultimately determine the clinical impact of transcriptomic biomarkers in global TB control efforts.

## 5 Conclusion

In conclusion, this comprehensive integrative analysis of multi-cohort transcriptomic data in tuberculosis represented a significant advancement in TB biomarker discovery, simultaneously delivering mechanistic insights into TB immunopathology and identifying validated, clinically applicable predictive biomarkers. Through our innovative combination of pathway-centric enrichment analysis, rigorous meta-analytical integration, and machine learning enhanced by explainable AI, we had successfully identified a conserved transcriptional signature of 347 core genes and three key immune pathways (NF-κB signaling, antigen presentation, TB-specific pathways) that transcend both technical platform variations and population heterogeneity—a critical achievement that has eluded previous single-cohort studies. Our findings not only confirmed and extended established paradigms of TB immunology but provide quantitative, genome-wide evidence at an unprecedented scale, with clear functional annotation linking observed signatures to known immunological processes. The exceptional diagnostic performance of our machine learning classifiers (mean AUC: 0.89 ± 0.04) across five independent cohorts, combined with the biological coherence of identified features, positions these findings for immediate clinical validation and eventual translation to point-of-care diagnostic platforms. The identified biomarkers, particularly those in interferon signaling (STAT1, IFITM1, ISG15) and antigen presentation (HLA-DRA, HLA-DRB1) pathways, represented high-priority targets for focused gene panel development and functional validation. Beyond TB, the analytical framework established here—integrating meta-analysis, pathway enrichment, and explainable machine learning—provided a generalizable template for biomarker discovery in other infectious diseases, inflammatory conditions, and complex diseases. As we enter an era of precision medicine, studies such as this that successfully bridge computational biology, immunology, and clinical medicine will be essential for advancing global health and achieving the WHO’s End TB Strategy goals of reducing TB incidence by 90% and deaths by 95% by 2035.

## Data Availability

The datasets generated during the study are available from the corresponding author upon reasonable request. The full trial protocol is also available from the corresponding author upon request. Requests to access these datasets should be directed to lt881117@163.com.
